# Beyond hemoglobin: Critical role of 2,3‐bisphosphoglycerate mutase in kidney function and injury

**DOI:** 10.1111/apha.14242

**Published:** 2024-10-18

**Authors:** Vera A. Kulow, Kameliya Roegner, Robert Labes, Mumtaz Kasim, Susanne Mathia, Claudia S. Czopek, Nikolaus Berndt, Philipp N. Becker, Gohar Ter‐Avetisyan, Friedrich C. Luft, Philipp Enghard, Christian Hinze, Jan Klocke, Kai‐Uwe Eckardt, Kai M. Schmidt‐Ott, Pontus B. Persson, Christian Rosenberger, Michael Fähling

**Affiliations:** ^1^ Charité—Universitätsmedizin Berlin, corporate member of Freie Universität Berlin and Humboldt‐Universität zu Berlin, Medizinische Klinik m.S. Nephrologie und Internistische Intensivmedizin (CCM) Berlin Germany; ^2^ Charité—Universitätsmedizin Berlin, corporate member of Freie Universität Berlin and Humboldt‐Universität zu Berlin, Institut für Translationale Physiologie (CCM) Berlin Germany; ^3^ Department of Molecular Toxicology German Institute of Human Nutrition Potsdam‐Rehbruecke (DIfE) Nuthetal Germany; ^4^ Deutsches Herzzentrum der Charité (DHZC), Institute of Computer‐assisted Cardiovascular Medicine Berlin Germany; ^5^ Charité—Universitätsmedizin Berlin, corporate member of Freie Universität Berlin and Humboldt‐Universität zu Berlin Berlin Germany; ^6^ Experimental and Clinical Research Center (ECRC) Charité—Universitätsmedizin Berlin, corporate member of Freie Universität Berlin and Humboldt‐Universität zu Berlin Berlin Germany; ^7^ Department of Nephrology and Hypertension Hannover Medical School Hannover Germany

**Keywords:** 2,3‐bisphosphoglycerate mutase (BPGM), acute kidney injury (AKI), glycolysis, inflammation, oxidative stress response, reactive oxygen species (ROS)

## Abstract

**Aim:**

2,3‐bisphosphoglycerate mutase (BPGM) is traditionally recognized for its role in modulating oxygen affinity to hemoglobin in erythrocytes. Recent transcriptomic analyses, however, have indicated a significant upregulation of BPGM in acutely injured murine and human kidneys, suggesting a potential renal function for this enzyme. Here we aim to explore the physiological role of BPGM in the kidney.

**Methods:**

A tubular‐specific, doxycycline‐inducible *Bpgm*‐knockout mouse model was generated. Histological, immunofluorescence, and proteomic analyses were conducted to examine the localization of BPGM expression and the impact of its knockout on kidney structure and function. In vitro studies were performed to investigate the metabolic consequences of *Bpgm* knockdown under osmotic stress.

**Results:**

BPGM expression was localized to the distal nephron and was absent in proximal tubules. Inducible knockout of *Bpgm* resulted in rapid kidney injury within 4 days, characterized by proximal tubular damage and tubulointerstitial fibrosis. Proteomic analyses revealed involvement of BPGM in key metabolic pathways, including glycolysis, oxidative stress response, and inflammation. In vitro, *Bpgm* knockdown led to enhanced glycolysis, decreased reactive oxygen species elimination capacity under osmotic stress, and increased apoptosis. Furthermore, interactions between nephron segments and immune cells in the kidney suggested a mechanism for propagating stress signals from distal to proximal tubules.

**Conclusion:**

BPGM fulfills critical functions beyond the erythrocyte in maintaining glucose metabolism in the distal nephron. Its absence leads to metabolic imbalances, increased oxidative stress, inflammation, and ultimately kidney injury.

## 1 INTRODUCTION

The Rapoport‐Luebering glycolytic shunt generates 2,3‐bisphosphoglycerate (2,3‐BPG)^1^ through the enzymatic action of 2,3‐bisphosphoglycerate mutase (BPGM) in erythrocytes.^2^ The primary recognized function of BPGM is to reduce the oxygen affinity to hemoglobin by 2,3‐BPG, thereby facilitating oxygen delivery to tissues. Although initially thought to be exclusive to erythrocytes, BPGM is highly conserved across evolution and is expressed in various organisms, including plants, fungi, and bacteria,^3^ suggesting a more general role. Supporting this, BPGM is expressed in the placenta,^4^ astrocytes,^5^ and tumor cells,^6^ where it has been associated with the regulation of glycolysis. Screening transcriptomic data of acutely injured murine kidneys following rhabdomyolysis^7^ retrieved BPGM as a prominently upregulated factor. This prompted us to seek for the location and function of BPGM within the kidney and potentially to discover the ancient role of this enzyme. The lack of a known transmembrane transporter for the highly polar 2,3‐BPG suggests that its action likely occurs within the producing cell. In line, 2,3‐BPG has been proposed to inhibit glycolysis.^5^, ^6^, ^8^ We, therefore, assumed that upregulation of BPGM in acute kidney injury (AKI) might be protective by moderating excessive glycolytic flux, especially under hypoxia or cellular stress. When the glycolytic pathway is upregulated, the increased flux of intermediates into other metabolic processes, such as the tricarboxylic acid (TCA) cycle, can overload the mitochondrial electron transport chain (ETC) or activate NADPH oxidase, both significant sites for reactive oxygen species (ROS) production.^9^ Oxidative stress poses potential harm to cellular components and plays a crucial role in the development of kidney diseases, including diabetic nephropathy.^10^, ^11^ Given that AKI combines hypoxic, oxidative, and inflammatory stress, we hypothesized that kidney BPGM could be renoprotective by balancing glucose metabolism and preventing ROS formation. Indeed, our observations indicated extensive cellular pathway modifications induced by BPGM, which are crucial for protective stress responses, as evidenced by the rapid development of spontaneous kidney injury in inducible tubular‐specific *Bpgm*‐knockout mice.

## 2 RESULTS

### 2.1 BPGM is constitutive to the distal nephron and upregulated in AKI

**FIGURE 1:**
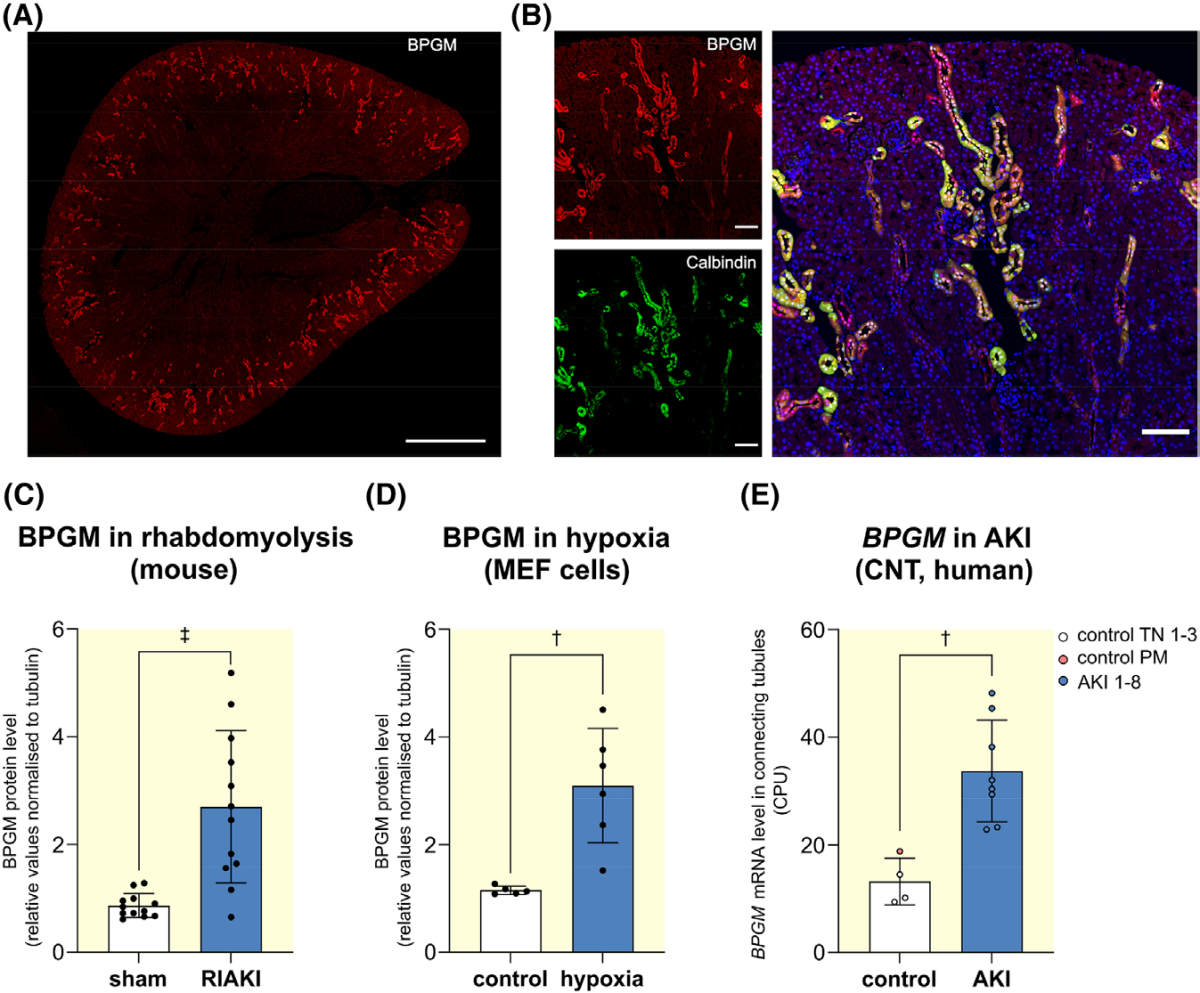
BPGM is expressed in renal tubular cells and is upregulated in AKI and hypoxia. (A) Immunofluorescence staining of BPGM (red) in mouse kidney section. Scale bar: 1000 μm. (B) Immunofluorescence staining of mouse kidney section showing co‐localization of BPGM (red) with calbindin (green) for DCT and CNT. Scale bar: 100 μm. For BPGM localization in further tubular segments, see Figure S1. (C) Upregulation of BPGM in kidneys of mice following 24 h of rhabdomyolysis‐induced AKI (*N* = 12), as described in Fähling et al.^7^ (D) Elevation of BPGM in mouse embryonic fibroblast (MEF) cells exposed to either hypoxia (1% O_2_) (*N* = 6) or control conditions (*N* = 5) for 24 h. (E) Plot displays statistical analysis of *BPGM* expression in connecting tubules (CNT) of control samples (*N* = 4; three tumor‐adjacent normal tissue samples [TN 1–3] and a post‐mortem biopsy from non‐AKI patient [control PM]). AKI samples (*N* = 8) result from post‐mortem biopsies from patients with AKI stage 2 or 3, as described in Hinze et al.^12^ Boxplots show the median with lower and upper quartile as box. Whiskers show the minimum and maximum values. Dots represent single values. Statistical analysis was performed using Student's *t*‐test.

We first observed renal BPGM in transcriptomic data obtained from rhabdomyolysis‐induced AKI.^7^ Confirmatory, immunofluorescence staining of BPGM revealed a predominantly tubular expression (Figure 1A). Proximal tubules (PT) lacked BPGM, as no co‐localization with megalin, a PT marker expressed in the brush border, was observed (Figure S1A). In contrast, BPGM co‐localized with markers for distal nephron segments: NKCC2 for thick ascending limbs (TAL, Figure S1B), NCC for distal convoluted tubules (DCT, Figure S1C), Calbindin for DCT and connecting tubules (CNT, Figure 1B), and Aquaporin‐2 for collecting ducts (CD, Figure S1D). BPGM expression was most pronounced in DCT and CNT. Supporting the transcriptomic data, BPGM protein level was upregulated in rhabdomyolysis‐induced AKI (Figure 1C), a model previously shown to cause kidney hypoxia.^7^ Hypoxia, a condition when oxygen demand exceeds supply, also caused BPGM upregulation in vitro using mouse embryonic fibroblast (MEF) cells after 24 h (Figure 1D). Further, *BPGM* was upregulated in CNT of human AKI samples, as shown by single‐cell RNA sequencing data obtained from kidney biopsies as described in Hinze et al.^12^ (Figure 1E). Similar to the mouse model, *BPGM* mainly distributed in the distal nephron (Figure S2A). Moreover, *BPGM* was upregulated especially in injured cells (Figure S2B,C). In urine samples from 32 AKI patients, as described in Klocke et al.,^13^ *BPGM* was elevated in cells derived from distal parts of the nephron and was enriched in cells that also express injury markers (Figure S3). Thus, BPGM is constitutively expressed in renal tubular cells of murine and human origin and is upregulated under conditions of hypoxia and AKI.

### 2.2 Inducible tubular Bpgm knockout causes AKI and fibrosis

**FIGURE 2:**
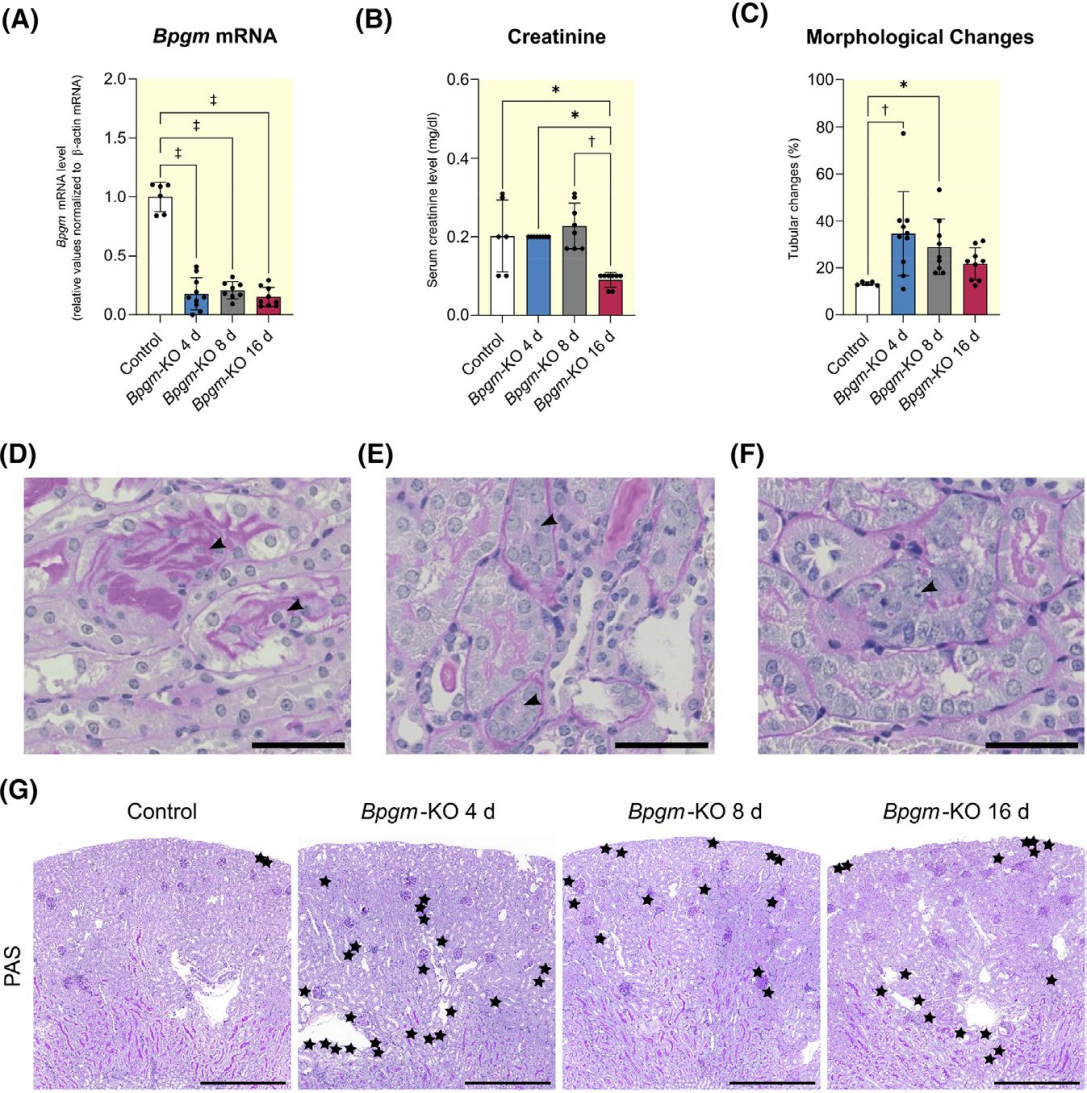
Inducible nephron‐specific *Bpgm* knockout leads to tubular injury. Inducible, Pax8‐driven tubular *Bpgm*‐KO was induced by doxycycline treatment 4, 8, and 16 d before kidney removal. Control mice received doxycycline for the given time point and were negative for *Cre*‐recombinase. (A) qPCR analysis for *Bpgm* confirms knockout. (B) Quantification of creatinine reveals a significant decline after 16 days of *Bpgm*‐KO. (C) Renal tubules of *Bpgm*‐KO mice show morphological changes associated with tubular injury as shown in (D–G). (D) Acute Tubular Necrosis. Arrowheads show tubules with necrotic changes including loss of brush border, cell disruption, and cell integrity loss. Scale bars: 50 μm. (E) Arrowheads show tubular basement membrane thickening. Scale bar: 50 μm. (F) Arrowhead shows a proximal tubule with loss of brush border and polyploidy cells. Scale bar: 50 μm. (G) Kidney PAS staining of control and *Bpgm*‐KO mice. Black stars indicate tubular morphological changes used for quantification of morphological changes in (C). Scale bars: 500 μm. Boxplots show the median with lower and upper quartile as box. Whiskers show the minimum and maximum values. Dots represent single values (control: *N* = 6; *Bpgm*‐KO 4 d: *N* = 10; *Bpgm*‐KO 8 d: *N* = 8; *Bpgm*‐KO 16 d: *N* = 9). Statistical analysis was performed using one‐way ANOVA or Kruskal‐Wallis test. Adjusted *P*‐values are shown.

**FIGURE 3:**
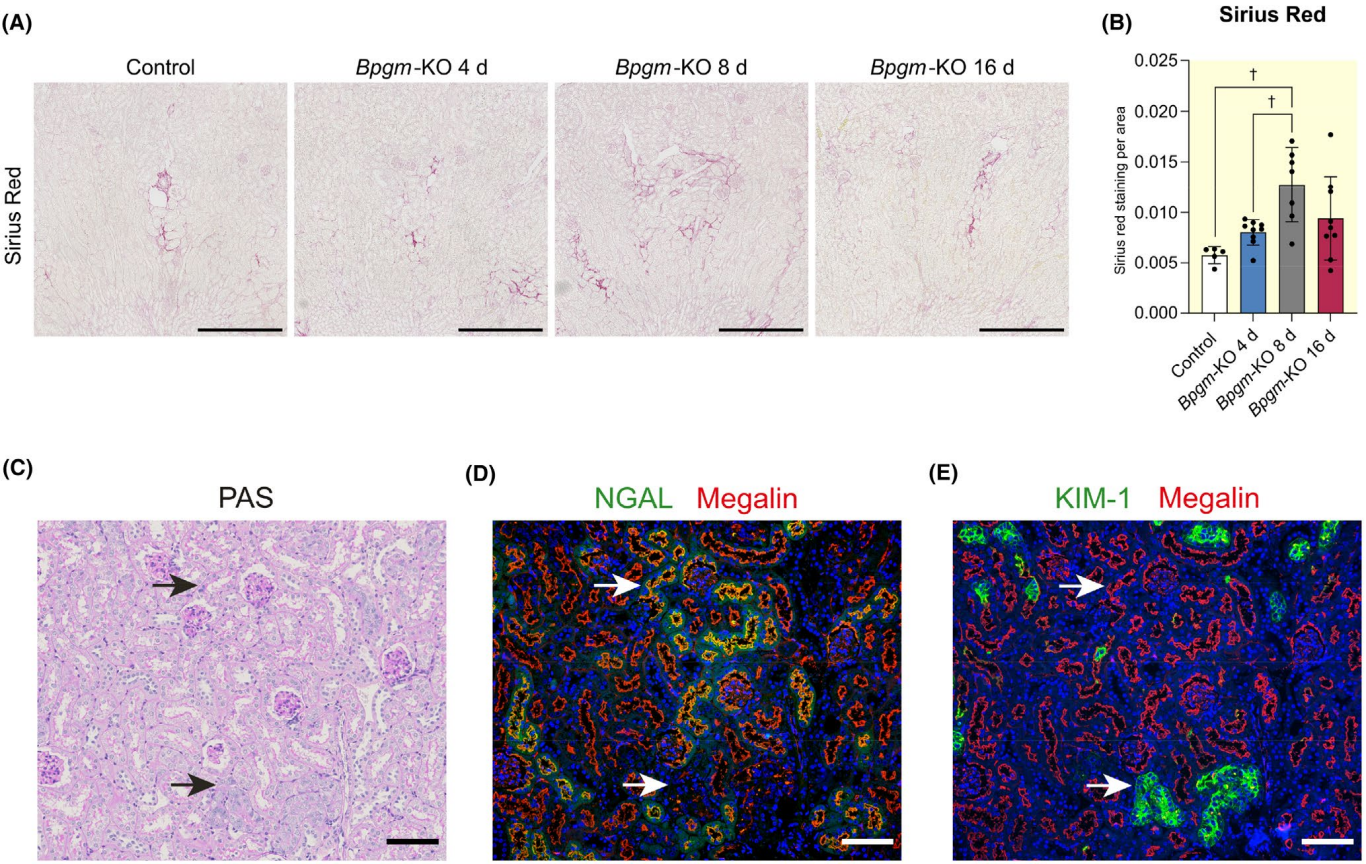
Inducible nephron‐specific *Bpgm* knockout leads to proximal tubular injury and fibrosis. (A) Picro‐Sirius Red staining of kidneys obtained from control and *Bpgm*‐KO mice (time course). Scale bar: 500 μm. (B) Quantification of Picro‐Sirius Red staining shown in (A). (C–E) Staining on parallel kidney sections of *Bpgm*‐KO mouse for PAS (C) and immunofluorescence double staining for megalin/ neutrophil gelatinase‐associated lipocalin (NGAL) (D) and megalin/kidney injury molecule (KIM)‐1 (E). Upper arrow: S1 segment of proximal tubule is NGAL‐positive and shows a preserved brush border and no overt damage on PAS, thus indicating mild damage. Lower arrow: S3 segment of proximal tubule is KIM‐1‐positive and shows partial loss of brush border and cellular congestion, thus indicating severe damage. Scale bars: 100 μm.

To study kidney BPGM function, we created a mouse model with doxycycline‐inducible, nephron‐specific conditional *Bpgm* knockout (*Bpgm*‐KO). After doxycycline injection, *Bpgm* mRNA was significantly reduced for at least 16 days (Figure 2A). Control mice received doxycycline accordingly but lacked *Cre* expression. *Bpgm*‐KO did not change creatinine levels up to 8 days, followed by a significant decline at day 16 (Figure 2B). Nevertheless, PAS staining in *Bpgm*‐KO indicated typical signs of nephron injury such as loss of brush border, cell disruption, cell integrity loss, tubular basement membrane thickening, and polyploidy of tubular cells (Figure 2C–F). On semi‐quantification, tubular damage was significant by days 4 and 8, but not by day 16 after induction of *Bpgm*‐KO (Figure 2C,H). Accordingly, renal fibrosis, which represents a hallmark in kidney diseases, was significant by day 8 and still detectable by day 16 of *Bpgm*‐KO (Figure 3A,B). To confirm rapid kidney injury following tubular loss of BPGM, we also tested for early AKI markers: kidney injury molecule‐1 (KIM‐1) and neutrophil gelatinase‐associated lipocalin (NGAL). By immunofluorescence staining, KIM‐1 and NGAL were undetectable in controls (Figure S4), but prominent in PT cells of *Bpgm*‐KO (Figure S5). Injured nephron portions were sharply delimitated. NGAL‐positivity occurred in S1 segments, while KIM‐1‐positivity occurred in S3 segments of PT cells (Figure 3C–E). The former was associated with mild, and the latter with severe tubular injury, as judged by PAS staining. As exemplarily shown for KIM‐1, early AKI markers were only enhanced at day 4 (Figure S6).

Together, our findings confirm that the loss of tubular BPGM rapidly causes renal damage. Tubular injury increases up to 8 days after induction of the *Bpgm*‐KO but seems to be compensated by cellular adaptation processes. Consequently, no further progression of the damage was observed after 16 days.

### 2.3 Bpgm‐knockout proteomics and pathway verification

**FIGURE 4:**
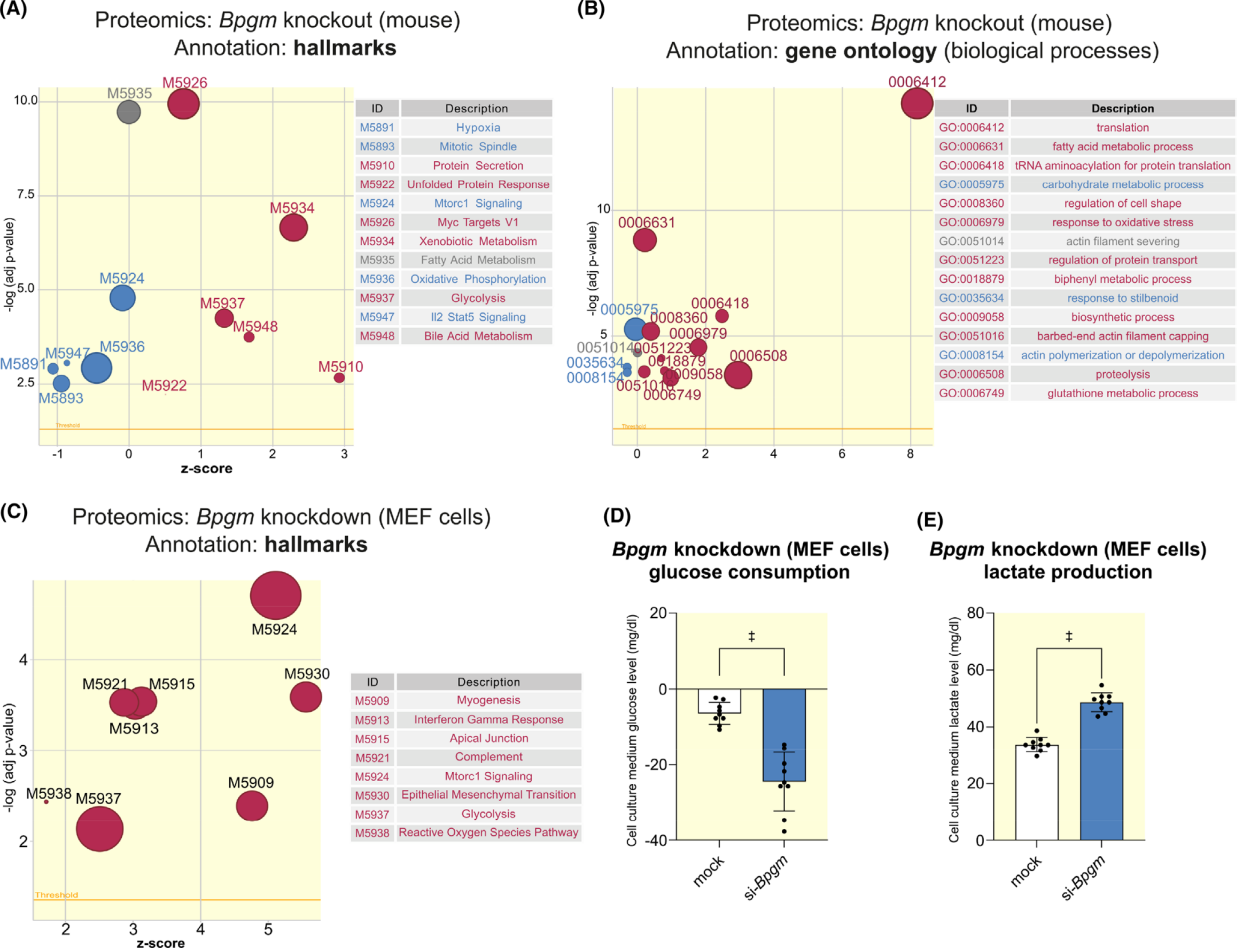
Proteomic analyses of mouse *Bpgm*‐KO (4 d) and cellular *Bpgm* knockdown reveal crucial BPGM functions in energy metabolism, oxidative stress, and immune response. (A, B) Proteomic analysis following 4 d of *Bpgm* knockout revealed 241 proteins affected by *Bpgm*‐KO. Gene set enrichment analysis indicated significantly regulated pathways either with the hallmarks (A) or gene ontology annotation (B). (C–E) MEF cells were transfected with siRNA suppressing *Bpgm* or mock siRNA for 48 h. (C) Bubble plots of proteomic analysis: *Bpgm* knockdown affected expression of 89 proteins. A negative Z‐score indicates that the majority of genes involved in the pathway are downregulated; a positive Z‐score indicates that the majority of genes involved in the pathway are upregulated. Glucose consumption (D) and lactate production (E) following 48 h of siRNA‐mediated *Bpgm* knockdown in MEF cells. Higher glucose consumption and lactate production rates following *Bpgm* knockdown confirm the in vivo findings that BPGM inhibits glycolysis. Boxplots show the median with lower and upper quartile as box. Whiskers show the minimum and maximum values. Dots represent single values (*N* = 9). Statistical analysis was performed using Student's *t*‐test.

To get more mechanistic insights into the function of BPGM in kidney tubular cells, we performed a proteomic analysis following 4 days of *Bpgm*‐KO. Gene set enrichment analyses using the annotations “hallmarks” (Figure 4A) and “gene ontology” (Figure 4B) revealed participation of BPGM in energy metabolism (“glycolysis,” “oxidative phosphorylation,” “fatty acid metabolism,” “Mtorc1 signaling,” “translation,” “hypoxia”), oxidative stress (“response to oxidative stress,” “glutathione metabolic process”) and modulation of immune response (“Il2‐Stat5 signaling”). A positive z‐score indicated that candidate genes of the pathway were predominantly upregulated.

**FIGURE 5:**
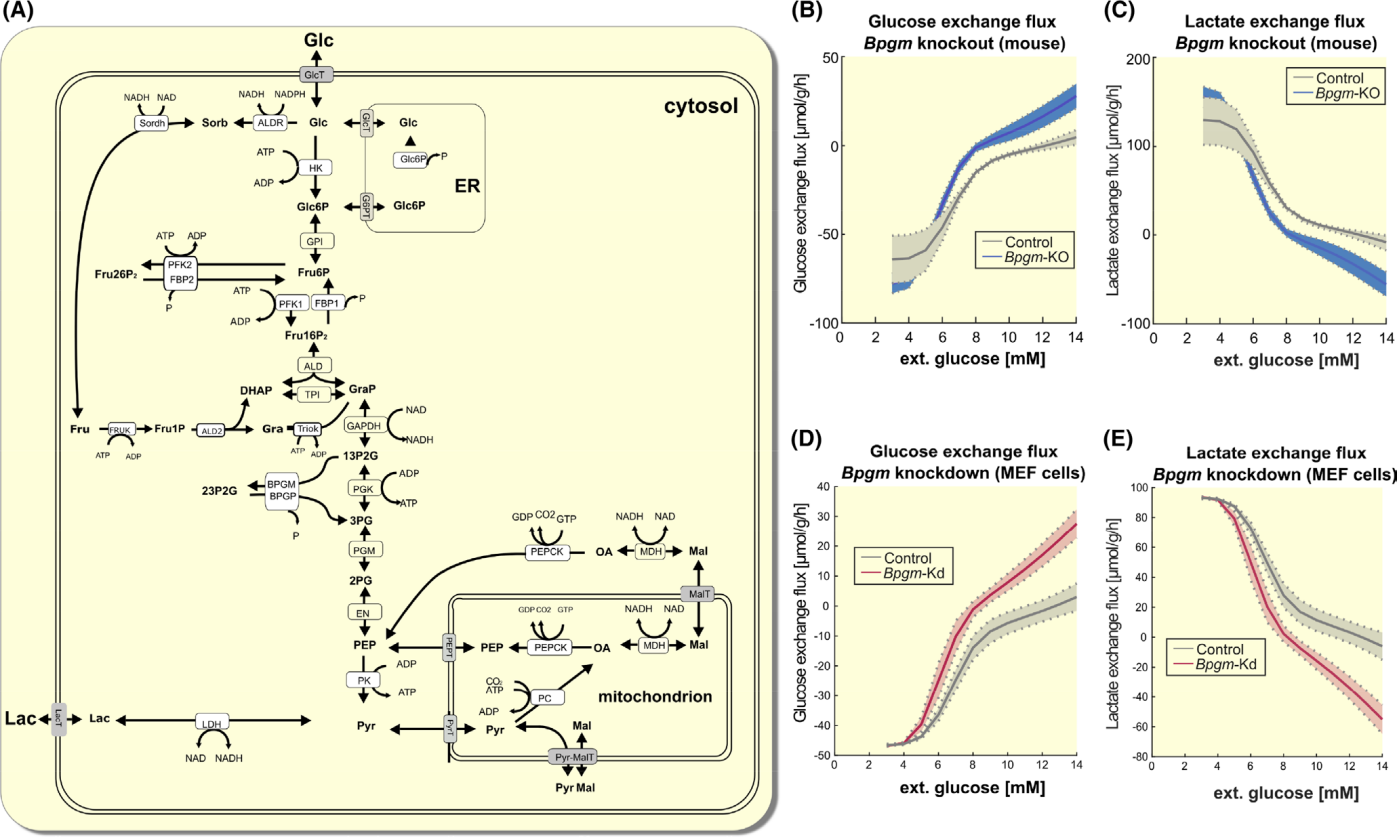
BPGM inhibits glycolysis. (A) Schematic representation of glucose metabolism. The model summarizes enzymes involved in glycolysis, gluconeogenesis, and the polyol pathway. Specifically, the model contains the following metabolites: Dihydroxyacetone phosphate (DHAP), fructose (Fru), fructose 1‐phosphate (Fru1P), fructose 6‐phosphate (Fru6P), fructose‐1,6‐bisphosphate (Fru16P2), fructose‐2,6‐bisphosphate (Fru26P2), glyceraldehyde 3‐phosphate (GAP), glucose (Glc), glucose 6‐phosphate (Glc6P), lactate (Lac), malate (Mal), oxaloacetate (OA), phosphate (P), phosphoenolpyruvate (PEP), 1,3‐bisphosphoglycerate (13P2G), 2,3‐bisphosphoglycerate (23P2G), 2‐phosphoglycerate (2PG), 3‐phosphoglycerate (3PG), pyrophosphate (PP), pyruvate (Pyr), and sorbitol (Sorb). The co‐factors NAD, its reduced form NADH, ADP, and ATP are not treated as dynamic variables. The physiological metabolic processes consuming Pyr during glycolysis are comprised of Lac formation and export. Kinetic rate laws of reaction rates are given in the Supplement section. Glucose (B) and lactate exchange flux (C) following *Bpgm*‐KO (red line) and control animals (blue line). Solid lines and shaded areas depict the mean and standard deviations of simulations for six individual proteomic data sets (as shown in Figure 4A,B) for each condition. Glucose (D) and lactate exchange flux (E) following *Bpgm* knockdown (red line) and mock transfection (green line) in MEF cells after 48 h. Solid lines and shaded areas depict the mean and standard deviations of simulations for six individual proteomic data sets (as shown in Figure 4C) for each condition. Data indicate an elevated rate of glycolysis under *Bpgm* deletion; thus, BPGM inhibits glycolysis.

As we used whole kidney tissue extracts for proteomic analysis, we aimed to reproduce findings at the cellular level. It turned out that MEF cells showed robust BPGM expression and are suitable for investigating the lack of BPGM on intracellular pathways. Proteomic data obtained from MEF cells following siRNA‐mediated *Bpgm* knockdown supported in vivo findings as they show regulation of similar pathways as in *Bpgm*‐KO: energy metabolism (“glycolysis,” “Mtorc1 signaling”), oxidative stress (“reactive oxygen species pathway”), and modulation of immune response (“interferon gamma response,” “complement”) (Figure 4C). Thus, MEF cells recapitulated crucial aspects of the pathways influenced by *Bpgm*‐KO in vivo and, therefore, provide a useful tool for specific pathway analyses in vitro. Supporting the finding that *Bpgm*‐KO causes elevated glycolysis, MEF cells showed higher rates of glucose consumption (Figure 4D) and lactate production (Figure 4E) following siRNA‐mediated *Bpgm* knockdown. To confirm our observations, we used a kinetic model of renal glucose metabolism (Figure 5A) that predicts the glycolytic and gluconeogenic capacities based on the data obtained from our in vivo and in vitro proteomic analyses. Indeed, modeling revealed that glucose consumption rate was significantly enhanced in *Bpgm*‐KO (Figure 5B), as was lactate production (Figure 5C), which was mirrored by modeling of MEF cell proteomic data (Figure 5D,E). Together, these data confirmed that BPGM inhibits glycolysis.

**FIGURE 6:**
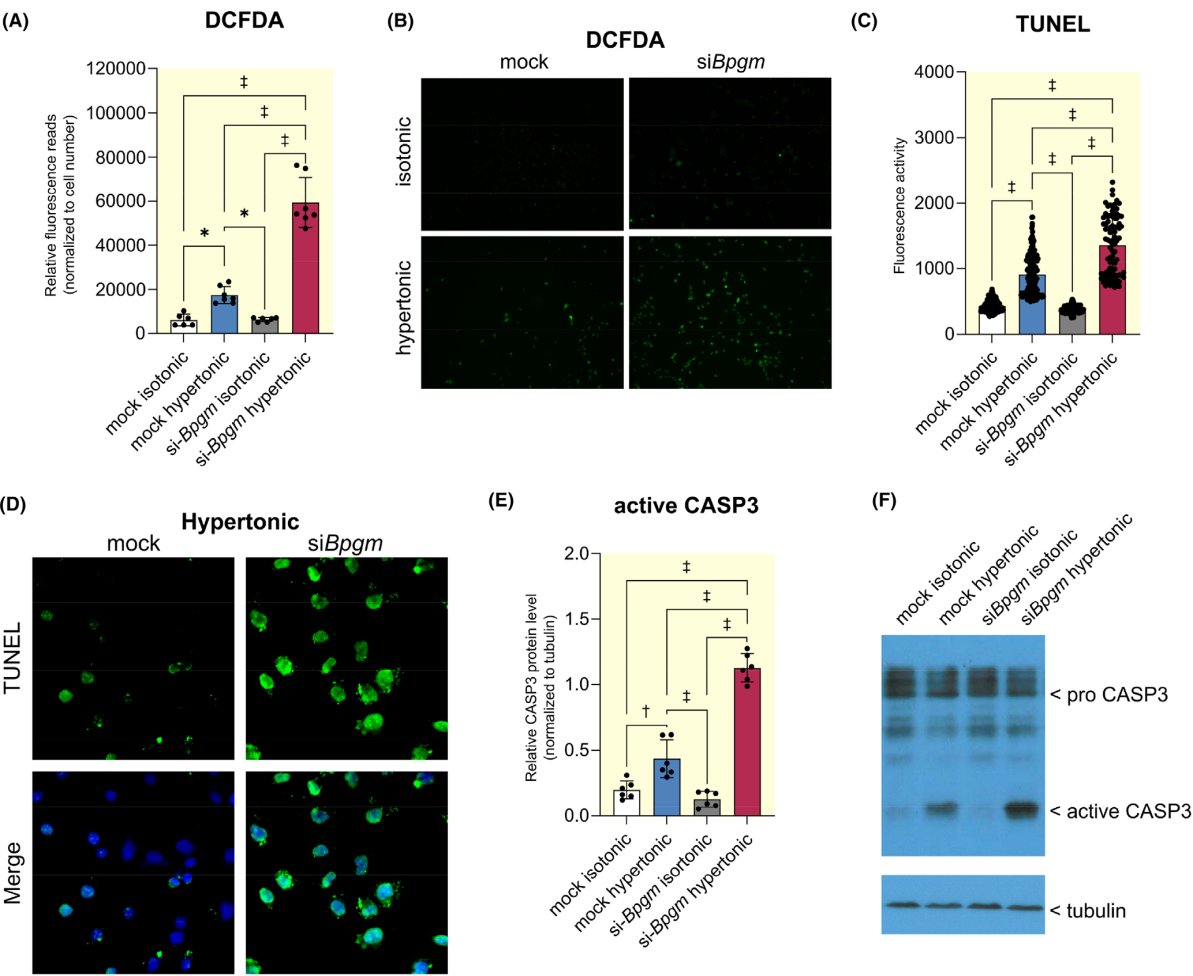
BPGM protects from ROS and apoptosis. MEF cells were transfected with siRNA suppressing *Bpgm* or mock siRNA for 24 h and exposed to plasma‐isotonic (280 mosm/L) or hypertonic (450 mosm/L) conditions for additional 24 h. (A) Analysis and (B) fluorescent microscopy pictures of DCFDA Assay that served for the detection of ROS following osmotic stress with or without *Bpgm* knockdown (*N* = 6). H_2_O_2_ was supplemented for 30 min in equal concentrations to reach detectable ROS levels. The loss of *Bpgm* reduced the ROS elimination capacity. (C) Analysis and (D) fluorescence microscopy pictures for apoptosis detection by TUNEL assay under hypertonic conditions. TUNEL staining is observed as fluorescent green. Osmotic stress caused elevated TUNEL signals per se that was significantly enhanced by *Bpgm* knockdown. Isotonic conditions yielded no obvious TUNEL signals (not shown). (E, F) Western blot analysis of the apoptosis marker active‐CASP3. Quantification (E) of active‐CASP3 (*N* = 6) reveals that *Bpgm* knockdown promotes elevated active‐CASP3 levels under osmotic stress that was further enhanced by *Bpgm* knockdown. A representative Western blot (F) is shown. Detection of tubulin served as loading control for normalization. Boxplots show the median with lower and upper quartile as box. Whiskers show the minimum and maximum values. Dots represent single values. Statistical analysis was performed using ordinary one‐way ANOVA analysis. Adjusted *p*‐values are shown.

Gene set enrichment analyses indicated that BPGM also plays a crucial role in the oxidative stress response. At the cellular level, siRNA‐mediated *Bpgm* knockdown per se did not yield elevated ROS level by using the cell‐permeant reagent DCFDA, a fluorogenic dye detecting hydroxyl, peroxyl, and other ROS activities (Figure 6A,B). Nevertheless, as proteomic data indicate the involvement of the glutathione system, we next subjected MEF cells to osmotic stress, a condition activating the polyol pathway that consumes high amounts of NADPH and disables the glutathione system. As a result, osmotic stress provokes ROS accumulation by alteration of glucose metabolism (Figure 6A,B). Indeed, under osmotic stress we observed a reduced ROS detoxification capacity by *Bpgm* knockdown (Figure 6A,B). Furthermore, *Bpgm* knockdown during osmotic stress was associated with a higher rate of apoptosis, as detected by TUNEL assay (Figure 6C,D) and the apoptosis marker active caspase‐3 (Figure 6E,F). Thus, the in vitro data confirm that BPGM helps to mitigate oxidative damage by regulating pathways that control ROS detoxification.

### 2.4 Bpgm‐KO: Interplay between nephron segments and immune cells

**FIGURE 7:**
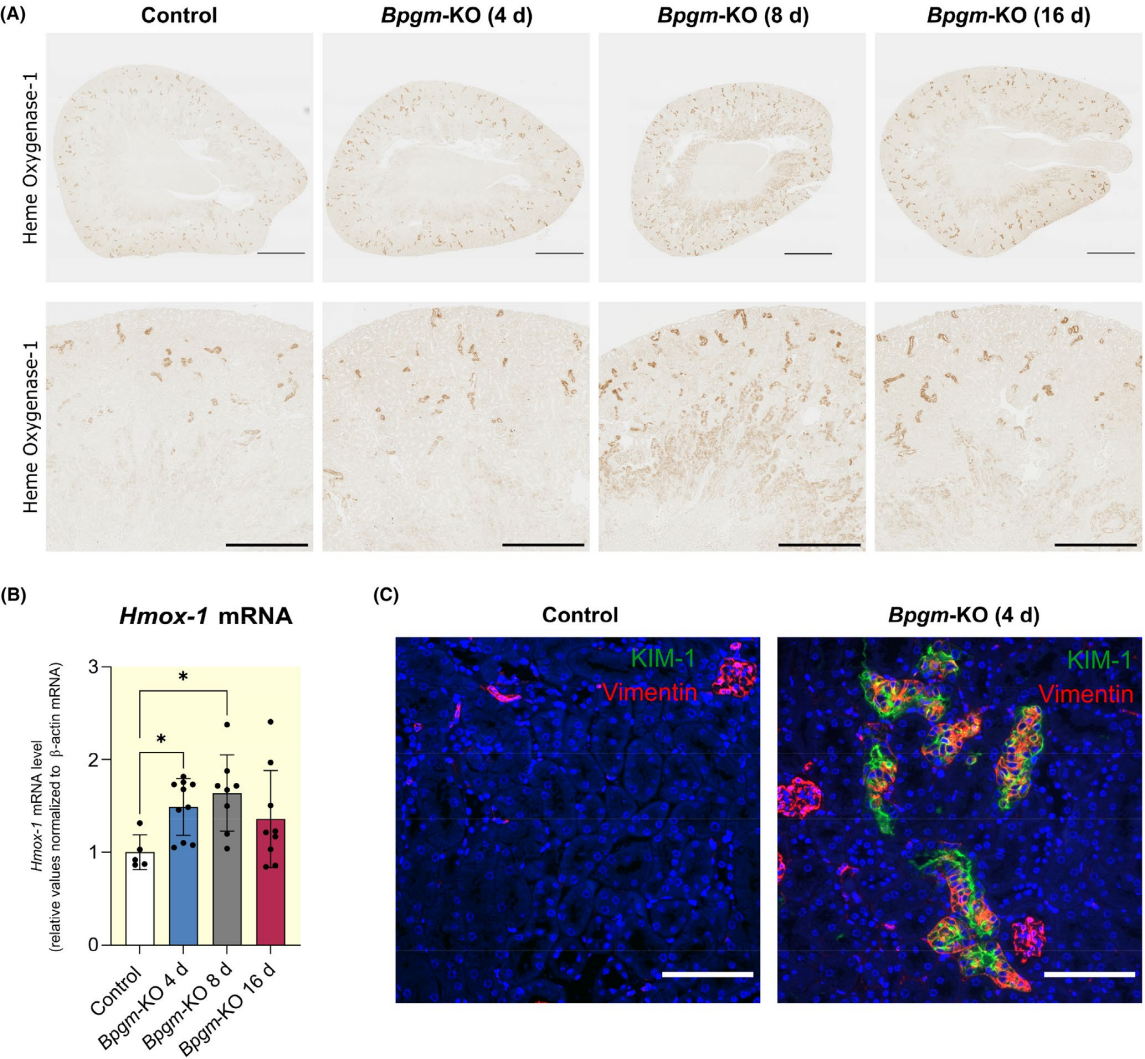
*Bpgm*‐KO leads to upregulation of hemoxygenase‐1, indicative of ROS signaling, and vimentin, indicative of cellular de‐differentiation. (A) Immunohistochemistry of hemoxygenase‐1 on *Bpgm*‐KO kidneys after 4, 8, and 16 days of knockout. Elevation of hemoxygenase‐1 confirms proteomic data (see Figure 4A,B). (B) qPCR analysis of *Hmox‐1* confirmed elevated expression. Whiskers show the minimum and maximum values. Dots represent single values (control: *N* = 5; *Bpgm*‐KO 4 d: *N* = 10; *Bpgm*‐KO 8 d: *N* = 8; *Bpgm*‐KO 16 d: *N* = 9). Statistical analysis was performed using one‐way ANOVA test. Adjusted *p*‐values are shown. (C) Kidney sections were double‐stained for the proximal tubular injury marker KIM‐1 (green) and the cellular *trans*‐differentiation marker vimentin (red). The expression level of vimentin in kidney tubular cells is notably higher in *Bpgm*‐KO mice compared to control mice. Co‐localization of KIM‐1 with vimentin indicates that injured tubular cells undergo de‐differentiation. Scale bars: 100 μm.

In vivo, activation of the ROS detoxification system is supported by elevation of hemoxygenase‐1 (*Hmox1*) (Figure 7A,B), a cell stress sensor rapidly induced after oxidative stress,^14^ with anti‐oxidative effects.^15^ Furthermore, adaptation to cell stress is mediated by several factors including nuclear factor erythroid 2‐related factor 2 (NRF2), which represents a transcription factor playing a key role in response to oxidative stress through activation of detoxifying enzymes, including Hmox1.^16^ Although NRF2 activation is mainly regulated by nuclear translocation, upregulation of *Nrf2* (Figure S7A) indicates adaptation to oxidative stress under prolonged *Bpgm*‐KO conditions. Supporting, *Keap1*, which binds NRF2 and inhibits its nuclear delivery, is significantly downregulated (Figure S7B).

Oxidative stress in turn is supposed to induce cellular *trans*‐differentiation that may help cells to survive. Acknowledged markers of cellular *trans*‐differentiation are vimentin and α‐smooth muscle actin (α‐SMA).^17^ Indeed, vimentin (Figure 7C) and α‐SMA (Figure S8) were strongly expressed in injured (KIM‐1 positive) proximal tubular cells. Notably, these data further confirmed that tubular injury mainly affects proximal tubules, while BPGM protein expression was detected in distal tubules.

**FIGURE 8:**
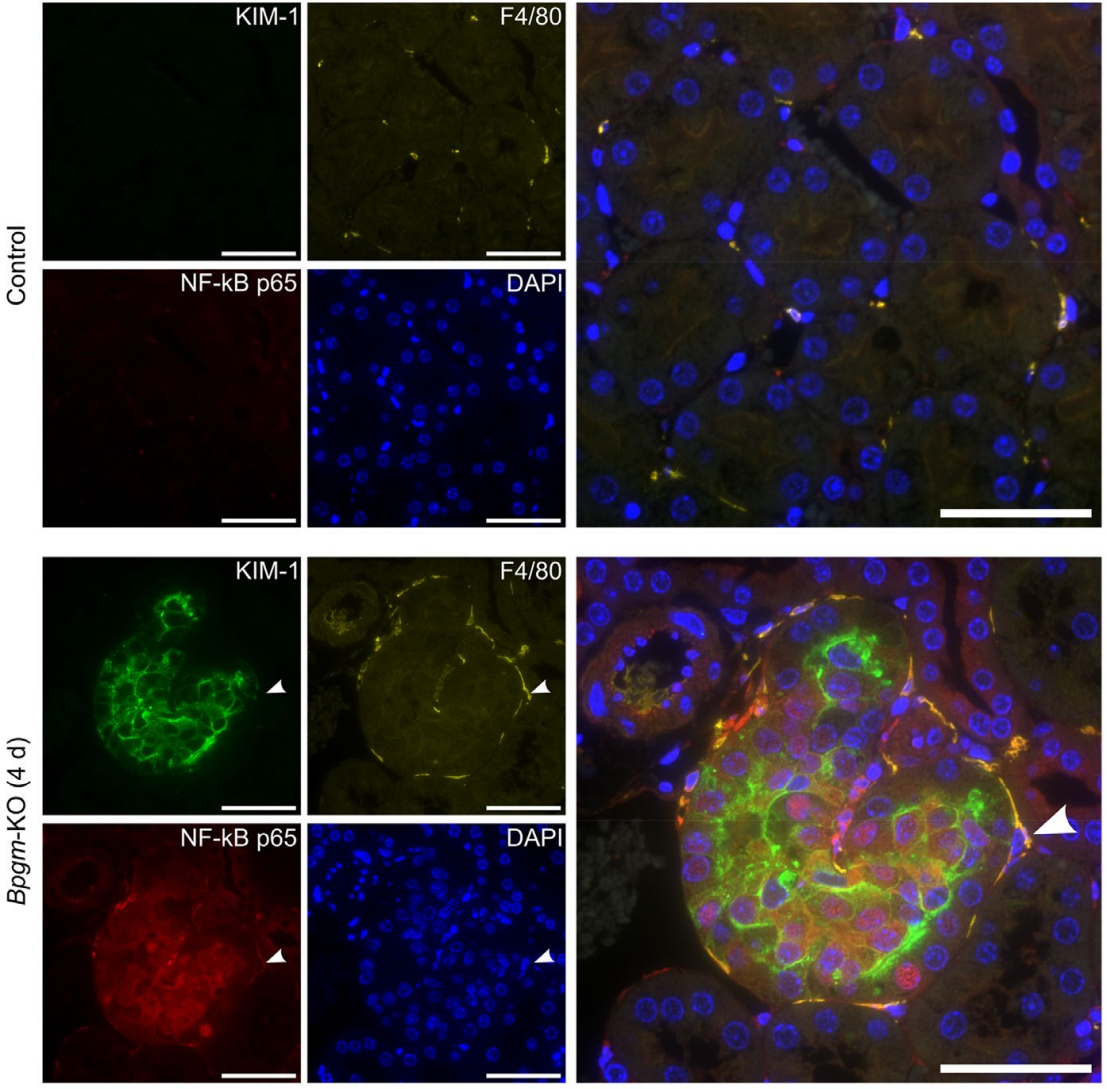
Inflammation and macrophages in injured tubules following *Bpgm* knockout. Immunofluorescence staining of *Bpgm*‐KO and control kidneys. The sections were stained for the specific proximal tubular injury marker KIM‐1 (green), F4/80 for macrophages (yellow), and Nf‐κB p65 (red). Injured tubules indicate elevated expression of regulatory p65 subunit of Nf‐κB, a key factor of inflammation. Further, macrophages surround the injured proximal tubule and are positive for Nf‐κB. Scale bars: 100 μm.

Obviously, tubular injury developed upstream of the distal nephron in which the *Bpgm* knockout has been induced. Remarkably, double immunofluorescence staining for KIM‐1 or NGAL and the distal tubular markers NCC and calbindin revealed the vicinity of injury and the *Bpgm*‐KO. These were within one to three tubular profiles distance (Figure S9). In search for a signal or messenger capable of bridging this gap, we first considered tissue resident macrophages. Our proteomic analysis indicated that *Bpgm*‐KO promotes cytokine pathways, which are known to prime macrophages. Indeed, following *Bpgm*‐KO, macrophages surround KIM‐1‐positive proximal tubules, whereas T cells or neutrophils do not (Figure S10). Moreover, detection of NF‐kB (nuclear factor of kappa‐B) regulatory p65 subunit, a key factor mediating inflammation that is activated by cytokines and ROS,^18^ revealed elevated nuclear staining in injured tubules and macrophages as a result of *Bpgm*‐KO (Figure 8).

Finally, renal fibrosis may result from inflammation and represents a hallmark in kidney diseases. Our observation of elevated fibrosis following *Bpgm*‐KO, which stagnated or even declined after 16 days, represents a strong indicator of regeneration from injury. *Ccl2* (C‐C motif chemokine ligand 2), a cytokine associated with fibrosis,^19^ is constantly upregulated in *Bpgm*‐KO (Figure S7C). In renal proximal tubules, ICAM‐1 has been shown to promote TGF‐α (transforming growth factor beta‐1) generation and fibrotic changes.^20^, ^21^ Consistent with Sirius red staining, *Icam1* is elevated up to 8 days of *Bpgm*‐KO, however, dropped to control level at Day 16 (Figure S7D). TNF‐α (tumor necrosis factor alpha), another pro‐inflammatory cytokine, is controversially discussed in the context of fibrosis.^22^ However, antagonistic effects to the master regulator of fibrosis, TGF‐α, are widely accepted. Notably, *Tnfa* was significantly upregulated at day 16 of *Bpgm*‐KO (Figure S7E), while *Tgfb* was first increased up to 8 days of *Bpgm*‐KO, but markedly declined at day 16 (Figure S7F). The latter finding aligns well with the observed stagnation of fibrosis after 16 days of *Bpgm*‐KO.

In sum, the knockout of *Bpgm* affects interactions between different nephron segments and immune cells. This suggests that BPGM may have a crucial role in maintaining the communication and signaling between various cell types in the kidney.

## 3 DISCUSSION AND CONCLUSIONS

Our study demonstrates that the acute depletion of BPGM in renal tubular cells triggers a detrimental cascade, culminating in tubular damage. We propose that the initial consequence of tubular *Bpgm* knockout is an exaggerated glycolytic response, leading to the accumulation of ROS and subsequent inflammation, collectively contributing to tubular injury. Our findings emphasize the critical role of renal BPGM in modulating glucose metabolism during stress, thereby protecting from ROS and inflammation.

Recent studies have highlighted a role of erythrocyte BPGM in the progression of chronic kidney disease by eAMPK‐dependent metabolic reprogramming and facilitated O_2_ delivery.^23^, ^24^ However, BPGM expression in renal tubular cells suggests it may have a distinct function. Initially, we hypothesized that tubular BPGM expression might allow 2,3‐BPG produced in the distal convoluted tubule to enter the vasa recta bloodstream, thereby reducing oxygen‐hemoglobin affinity and enhancing oxygen delivery to the TAL in the outer medulla. However, extensive research did not support this notion. Instead, we discovered an intracellular role for BPGM in inhibiting glycolysis, likely through interactions with hexokinase^8^ and PGAM‐1,^25^ thereby modulating glucose metabolism.

Dysregulated glycolysis, as seen in *Bpgm*‐KO, is linked to oxidative stress in various forms of kidney injury,^26^, ^27^ most notably diabetic nephropathy—the leading cause of end‐stage renal disease.^28^ Beyond the direct effects of 2,3‐BPG and ROS signaling, glycolytic metabolites activate alternative pathways, such as serine synthesis,^25^ potentially facilitating broad alterations in cellular adaptations. This may explain the extensive proteomic changes observed following tubular *Bpgm*‐KO. Moreover, disturbances in glucose metabolism, particularly those impacting the NADPH‐consuming polyol pathway and the NADPH‐producing pentose phosphate pathway,^29^ can disrupt cellular redox equilibrium by altering NADPH availability. NADPH is crucial for the glutathione system, the primary cellular defense against ROS.^30^ Thus, our findings highlight BPGM as a novel key factor in renal signaling and underscore the importance of glucose metabolism for kidney homeostasis.

Interestingly, BPGM expression is limited to a relatively small population of kidney cells in the distal nephron. Remarkably, the most evident injury occurs in the proximal tubule, which lacks significant BPGM expression and is located upstream. The absence of BPGM expression in proximal tubules is unsurprising, given their critical role in gluconeogenesis and the minimal expression of glycolytic enzymes.^31^, ^32^ Nevertheless, it should be noted that we cannot exclude the possibility that BPGM is expressed at low, yet functionally important levels in PT cells, as human single‐cell sequencing data also indicate *BPGM* mRNA in PT‐cell clusters.

We propose three possible explanations for the distal‐to‐proximal crosstalk phenomenon: (1) BPGM activity in the distal nephron may help sustain signaling toward the proximal tubule, and the loss of these signals could have consequences; (2) the absence of BPGM could generate harmful signals directed at the proximal tubule; or (3) the lack of BPGM may increase oxygen consumption in the distal tubules, turning them into oxygen sinks. As a result, the neighboring proximal tubules, which are limited in their capacity for anaerobic glycolysis, could suffer hypoxic injury. Our proteomic analyses suggest that *Bpgm* loss activates pathways related to interleukin and interferon signaling, complement system, as well as ROS, all of which are known to prime macrophages.^33^ Therefore, we hypothesize that macrophages may play a key role in signal transmission. In healthy kidneys, macrophages are distributed in defined patterns, act as sentinels, and regulate tissue homeostasis.^34^ Macrophages, therefore, are prime candidates for messengers between different nephron segments. Indeed, following *Bpgm*‐KO in vivo, macrophages were observed surrounding KIM‐1‐positive proximal tubules. However, since proximal tubular injury was already present at the earliest time point investigated, macrophages may be either the cause or consequence of such tubular injury. We assume that macrophages could have been activated by ROS or cytokines^35^ released from DCT cells lacking BPGM, subsequently leading to PT‐cell injury. Alternatively, Humphreys et al. showed that KIM‐1 recruits macrophages to the renal tubular interstitium.^36^ Thus, following *Bpgm* knockout, KIM‐1 may have attracted surrounding macrophages. Considering the latter aspect, an alternative hypothesis could suggest that the distal convoluted tubule may also serve to supply glycolytic metabolites to the proximal tubule, which itself lacks glycolytic activity. In this scenario, glycolytic activity in the DCT could be linked to the glomerular filtration rate (GFR) and the need for salt reabsorption or hormonal regulation (e.g., aldosterone). A dysregulation of glycolysis in the DCT could therefore directly impact the metabolism of the proximal tubule cells.

In AKI, PT cells can switch to a dedifferentiated and proliferative phenotype, enabling regenerative capacity following injury.^37^ A hallmark of these cells is KIM‐1 expression, which binds apoptotic cell fragments to clear debris from the tubular lumen.^38^ Injured proximal tubule cells that fail to undergo normal repair develop a proinflammatory and profibrotic phenotype promoting chronic kidney disease.^39^ As we found KIM‐1‐positive PT cells alongside fibrosis, our *Bpgm*‐knockout model indeed resembles subacute kidney injury. Remarkably, our findings reveal that tubular injury diminishes and fibrosis stagnated 16 days after *Bpgm*‐KO, indicating the regenerative capacity of kidneys. However, the drop in creatinine level observed at this stage may be attributed to hyperfiltration, a potential precursor to future kidney function decline. We hypothesize that tubular cells adopt a new expression pattern, ensuring kidney survival but altering their ability to perform physiological functions and adapt to stress. These insights highlight the need for further research to uncover the long‐term implications of *Bpgm*‐KO.

While increased serum creatinine levels become evident only when kidney function falls below 50%, the significance of early AKI markers and activated pathways within an intervention‐applicable timeframe cannot be overstated. Our investigation has unveiled an unexpected role of BPGM in preserving renal well‐being. Thus, our model of tubular *Bpgm*‐KO will help identify early AKI pathways and mechanisms, providing valuable insights into potential intervention strategies.

## 4 MATERIALS AND METHODS

### 4.1 Study approval

Local authorities (Landesamt für Gesundheit und Soziales, Berlin: G0198/18) approved all studies that were conducted according to American Physiological Society guidelines.

### 4.2 Generation of Bpgm‐knockout mouse

*Bpgm*‐knockout mice were generated by crossbreeding double transgenic Pax8‐rtTA/LC1 mice^40^ and mice homozygous for the floxed *Bpgm* allele. In detail, mice with a floxed *Bpgm* allele were generated by using cryopreserved sperm of *Bpgm* ^tm1a^(KOMP)Wtsi (ESC clone ID; EPD0190_5_G03, Sanger Institute) mice and female C57BL/6‐Tg(CAG‐Flpe)2Arte mice. The offspring were then crossed with Pax8‐rtTA/LC1 mice to obtain mice carrying the floxed *Bpgm* allele and the Pax8‐rtTA and LC1 alleles (Pax8‐rtTA/LC1/*Bpgm* ^+/flox^). By further inbreeding, mice homozygous for the floxed *Bpgm* allele and carrying the Pax8‐rtTA and LC1 alleles were generated. These mice had a mixed background (C57BL/6N / C57BL/6N Tac) and were compared to littermates. The *Cre*‐mediated deletion of *Bpgm* can be induced by doxycycline and is restricted to tubular cells of the kidney.

### 4.3 Animal experiments

Male and female mice (18–30 g, 11 weeks old) were fed a standard rodent chow and water ad libitum. To achieve *Bpgm* deletion, doxycycline (100 mg/kg BW) was injected *i.p*. Mice not expressing Cre after doxycycline injection served as controls. Kidneys were removed after 4, 8, and 16 days. Animal experiments on rhabdomyolysis‐induced AKI were described in Fähling et al.^7^

### 4.4 Cell culture experiments

Mouse embryonic fibroblast (MEF) cells were found to show robust BPGM expression and, thus, are suitable for in vitro analysis of BPGM expression. MEF cells (ATCC‐No.: #SCRC‐1008, RRID: CVCL_9115) were cultured under sterile conditions at 37°C, 95% air, and 5% of carbon dioxide, using RPMI‐1640 Medium (#R0883, Sigma‐Aldrich, USA), supplemented with 10% (v/v) fetal bovine serum (#S0115, Biochrom GA, Germany), 1% (v/v) penicillin‐streptomycin (10,000 U/mL, #15140–122, Thermo Fisher Scientific, USA), and 1% (v/v) L‐Glutamine solution 200 mM (#G7513, Sigma‐Aldrich, USA).

Knockdown of target genes was performed by transfecting cells with ON‐TARGETplus mouse *Bpgm* siRNA (#L‐058581‐01‐0005) or ON‐TARGETplus non‐targeting control pool (mock, #D‐001810‐10‐20) at a final concentration of 25 nM using DharmaFECT 1 (#T‐2001‐07A, Horizon Discovery Ltd., UK) according to the manufacturer's instructions. ON‐TARGETplus SMARTpool siRNA sequences are shown in Table S1.

Overexpression of BPGM was performed by transfection with Myc‐DDK‐tagged human *BPGM* plasmid (#RC202105, OriGene Technologies, Inc., USA), using ROTIFect transfection reagent (#P001.4, Carl Roth GmbH + Co. KG, Germany), according to the manufacturer's recommendation. The plasmid concentration equaled 0.5, 1, and 2 μg. An equal amount of cloning vector PCMV‐XL5 (#PCMV6XL5, OriGene Technologies, Inc., USA) served as mock transfection.

To increase the osmolarity of medium up to 450 mosm/L, 0.2 M sucrose was added to the cell culture medium. Measurement of control medium indicated an osmolality of 280 ± 10 mosmol/L. For hypoxic conditions, cells were placed in a Whitley H35 Hypoxystation (Don Whitley Scientific), where oxygen was replaced by nitrogen (1% O_2_, 5% CO_2_, 37°C). Cells cultured under normoxia (21% O_2_, 5% CO_2_, 37°C) served as controls. After 24 h under hypertonic or hypoxic conditions, cells were washed two times with ice‐cold PBS, sedimented, and resolved with either RNA‐STAT‐60 (Cat. # CS‐502, Tel‐Test Inc., USA) for RNA isolation or lysis buffer (50 mM Tris pH 6.8, 4 M urea, 1% SDS, and 12.5 mM DTT) for Western blot analysis.

### 4.5 Glucose and lactate measurement

Cell culture supernatants were collected and centrifuged at 1.000 rpm for 1 minute at RT. Lactate and glucose were measured using an ABL800 Flex PLUS Radiometer (Radiometer GmbH, Germany).

### 4.6 TUNEL assay

TUNEL (terminal deoxynucleotidyl transferase dUTP nick end labeling) staining was performed using an In Situ Cell Death Detection Kit, Fluorescein (Cat. # 11684795910, Roche, Switzerland) according to the manufacturer's protocol. A Hoechst 33342 solution was used for nuclear counterstaining (Cat. #62249, Thermo Scientific, USA). The stained slides were observed and photographed with an Eclipse Ti2‐A microscope, DS‐Ri2 camera and NIS‐Elements software (Nikon, USA).

### 4.7 ROS measurement—DCFDA assay

Cells were exposed to either control or hypertonic conditions for 24 h, followed by treatment with 10 μM 2′,7′–dichlorofluorescein diacetate (Cat. #D6883, DCFDA, Sigma‐Aldrich) in serum‐free media for 30 min in the dark at 37°C and 5% CO_2_. Cells were washed with PBS and allowed to recover in the presence of 0.03% H_2_O_2_ for 30 min at 37°C and 5% CO_2_. Fluorescence intensity was read on a Synergy HTX multimode plate reader (BioTek Instruments GmbH). For each condition, 4 separate samples were used for cell counting. Fluorescence reads were normalized to cell number.

### 4.8 Quantitative polymerase chain reaction

qPCR was performed as recently described,^41^ and results were analyzed using the ΔΔCt method.^42^ Each sample was analyzed in triplicates, and their arithmetic means were normalized against the housekeeping gene β‐actin. Primer sequences are shown in Table S2.

### 4.9 Western blotting

Western blotting was performed as described.^41^ Antibodies used are listed in Table S3. Intensities of chemiluminescence signals were quantified using Image Studio Lite Version 5.2 Software (LI‐COR Biosciences Inc., USA). Protein levels were normalized to tubulin (TUBB2B). Verification of anti‐BPGM antibody is shown by siRNA‐mediated knockdown (Figure S11A,C) and overexpression of a Myc‐DDK‐tagged BPGM (Figure S11B,D).

### 4.10 Blood parameters

Plasma creatinine was measured by Labor Berlin—Charité Vivantes GmbH (Berlin, Germany).

### 4.11 Histological analysis

Paraffin‐embedded tissues were sliced into 1.5 μm (Immunofluorescence, Immunohistochemistry and PAS) or 4 μm (Sirius Red) thin sections and incubated for 16 h at 60°C to melt excessive paraffin. Deparaffinization was achieved using xylene followed by rehydration through decreasing ethanol solutions and *Aqua bidest*. Stained slices were recorded through an Eclipse Ti2‐A microscope and a DS‐Ri2 camera controlled by the NIS‐Elements software (Nikon, USA).

### 4.12 Immunofluorescence staining (IF) and immunohistochemistry (IHC)

For IF and IHC, rehydrated slices were pressure‐cooked for 12 minutes in 1x Target Retrieval Solution (Cat. #S1699, Agilent Technologies, Inc., USA) and unspecific proteins were blocked for 1 h at RT with either 5% skimmed milk in TBS‐T (IF) or RTU horse serum (IHC; Cat. #PK‐7800, Vector Laboratories, USA). Primary antibodies were diluted in Antibody‐Diluent (IF; Cat. #S3022, Agilent Technologies, Inc., USA) or RTU horse serum (IHC) and incubated overnight at 4°C. After 3 washing steps in TBS‐T, the appropriate secondary antibody was applied for 1 h at RT. For immunohistochemistry, slices were additionally incubated with DAB (Cat. #SK‐4100, Vector Laboratories, USA). Slices were mounted using Immu‐Mount™ (Cat. #9990402, Thermo Fisher Scientific Inc., USA). Used antibodies are listed in Table S3.

### 4.13 Periodic acid‐Schiff (PAS) and Sirius Red staining

Rehydrated slices were stained with the PAS‐staining kit (Cat. #12153.00500, Morphisto GmbH, Germany) or Sirius Red staining Kit (Cat. #13425.00250, Morphisto GmbH, Germany) according to the manufacturer's protocol. Stained slices were dehydrated and mounted with a synthetic mounting medium (Roti®Histokitt II, Cat. #T160.1, Carl Roth GmbH, Germany).

### 4.14 Proteome analysis by DIA LC–MS and DIA‐NN

Proteomic analysis was carried out by the Core Facility High‐Throughput Mass Spectrometry at Charité‐Universitätsmedizin Berlin. Sample preparation was performed as described in Müller T et al.^43^ Briefly, after peptide determination analysis was performed by LC–MS/MS. Raw data were processed using DIA‐NN 1.8^44^ with scan window size set to 11 and MS2 and MS1 mass accuracies set to 20 and 10 ppm, respectively. A spectral library free approach and mouse UniProt (UP000000589, Reviewed, Canonical, downloaded 2021‐01‐27)^45^ were used for annotation. DIA‐NN used a filter of 1% FDR on peptide level. Whole kidney lysates were used for the proteomic analysis of kidney samples.

Differential expression was analyzed with the DEP^46^ package for R (version 3.6.2, R Core Team, 2019). Data were filtered (threshold = 2), normalized by variance stabilizing transformation, and imputed with the quantile regression‐based left‐censored function.

### 4.15 Modeling renal glucose metabolism

The kinetic model is based on a previously published model of glucose metabolism^47^ and HEPATOKIN1,^48^ encompassing glycolytic, gluconeogenic, and polyol pathways. Time‐dependent variations of model variables (i.e., concentration of metabolites and ions) are governed by first‐order differential equations. Numerical values for kinetic parameters of the enzymatic rate laws derived from reported kinetic studies of the enzymes and are provided at the end of the Supplemental Information section.

Individual instantiations of the model were generated using the protein intensity profiles from quantitative shotgun proteomics to scale the maximal activities of enzymes and transporters, thereby exploiting the fact that the maximal activity of an enzyme is proportional to the abundance of the enzyme protein according to the relation:

$$v_{\max}^{\text{sample}}=v_{\mathrm{mx}}^{\text{mean control}}\frac{E^{\text{sample}}}{E^{\text{mean control}}}$$

*E* ^mean control^ denotes the mean protein abundance in the control group, and *E* ^sample^ denotes the protein abundance of enzyme *E* in a sample. For a detailed description, see Berndt et al.^48^

### 4.16 Gene set enrichment analysis

The SetRank package^49^ for R was used. The analysis was performed as recommended with the hallmark annotation tables from MSigDB.^50^ All mapped proteins were used as background set. For building the set collection, maxSetSize of 500 was used. SetRank analysis was performed with ranks and an FDR cutoff of 0.01. Data were visualized using the GOplot package for R.^51^

### 4.17 Statistics

The GraphPad Prism software (Version 8, USA) was used for statistical analysis. Outliers were identified by the ROUT method (*Q* = 5%).^52^ The Kolmogorov‐Smirnov test assessed normal distribution. For comparison of 2 groups, Student's *t*‐test (normal distribution) or Mann‐Whitney test (no normal distribution) was used. For more than 2 groups in normal distributed data with equal standard deviation (largest SD difference <twofold), the ordinary one‐way ANOVA followed by Tukey's post‐hoc test was used. If equal standard deviation could not be assumed (largest SD difference >twofold), the Brown‐Forsythe ANOVA was used, followed by Dunnett's T3 post‐hoc test. Non‐parametric Kruskal‐Wallis test with Dunn's post‐hoc test analyzed data without normal distribution. For dose‐response curves, non‐linear regression was performed with a second‐order polynomial least square fit. The extra sum‐of‐square F test determined significant differences between curves. *p*‐values below 0.05 were considered significant.

## AUTHOR CONTRIBUTIONS

**Vera A. Kulow:** Investigation; writing – original draft; methodology; validation; visualization; writing – review and editing; formal analysis; data curation. **Kameliya Roegner:** Investigation; methodology; validation; formal analysis. **Robert Labes:** Investigation; methodology; validation; software; formal analysis; visualization. **Mumtaz Kasim:** Investigation; methodology; validation; formal analysis. **Susanne Mathia:** Investigation; methodology; validation; formal analysis. **Claudia S. Czopek:** Investigation; validation; methodology. **Nikolaus Berndt:** Investigation; methodology; validation. **Philipp N. Becker:** Investigation; methodology; validation. **Gohar Ter‐Avetisyan:** Investigation; methodology; validation. **Friedrich C. Luft:** Writing – original draft; writing – review and editing; formal analysis. **Philipp Enghard:** Investigation; validation; formal analysis; methodology. **Christian Hinze:** Investigation; validation; formal analysis; methodology. **Jan Klocke:** Investigation; validation; methodology; formal analysis. **Kai‐Uwe Eckardt:** Writing – review and editing; formal analysis. **Kai M. Schmidt‐Ott:** Investigation; validation; formal analysis; methodology. **Pontus B. Persson:** Writing – review and editing; formal analysis. **Christian Rosenberger:** Funding acquisition; writing – original draft; investigation; writing – review and editing; project administration; supervision; resources; formal analysis; data curation. **Michael Fähling:** Conceptualization; investigation; funding acquisition; writing – original draft; methodology; validation; visualization; writing – review and editing; formal analysis; project administration; data curation; supervision; resources.

## FUNDING INFORMATION

This study was funded by Deutsche Forschungsgemeinschaft (DFG, German Research Foundation): Project ID 394046635, SFB 1365 (to M.F. and C.R.) and Deutsche Forschungsgemeinschaft: Project ID 538820275, FA 845/7‐1 (to M.F.). NB acknowledges support by the German Federal Ministry of Education and Research (BMBF) as part of the LiSyM‐Cancer program (DEEP‐HCC; grant no. 031L0258H and 031L0315G).

## CONFLICT OF INTEREST STATEMENT

The authors have declared that no conflict of interest exists.

## Supporting information


Figure S1:
Figure S2:
Figure S3:
Figure S4:
Figure S5:
Figure S6:
Figure S7:
Figure S8:
Figure S9:
Figure S10:
Figure S11:
Table S1:
Table S2:
Table S3:

## Data Availability

All data reported in this paper will be shared by the lead contact upon request. Proteomic data have been deposited at PRoteomics IDEntifications Database (PRIDE, *Bpgm*‐knockout mice data: PXD040789; *Bpgm* knockdown in MEF cells: PXD033095; http://www.ebi.ac.uk/pride) and are publicly available as of the date of publication.
