# Supplementary material for: Beyond hemoglobin: Critical role of 2,3‐bisphosphoglycerate mutase in kidney function and injury
Source: Acta Physiol (Oxf). 2024 Oct 18;241(1):e14242. doi: 10.1111/apha.14242 (PMC11674375; doi:10.1111/apha.14242)
Supplement: Supplementary file 1 — Figure S1: Figure S2: Figure S3: Figure S4: Figure S5: Figure S6: Figure S7: Figure S8: Figure S9: Figure S10: Figure S11: Table S1: Table S2: Table S3: [file APHA-241-e14242-s001.pdf]

SUPPLEMENTAL MATERIAL

OVERVIEW

- Supplemental Table 1: ON-TARGETplus SMARTpool siRNA Sequences.
- Supplemental Table 2: List of primers used for qPCR.
- Supplemental Table 3: List of antibodies used.
- Supplemental Figure 1: BPGM localization in the kidney.
- Supplemental Figure 2: Single-cell transcriptomic data from human AKI kidney biopsies.
- Supplemental Figure 3: Expression of *BPGM* in urine derived human kidney tubular epithelial cells following AKI.
- Supplemental Figure 4: Immunofluorescence staining of kidney injury markers KIM-1 and NGAL following 4 days of *Bpgm*-KO.
- Supplemental Figure 5: Tubular localization of kidney injury markers KIM-1 and NGAL following 4 days of *Bpgm*-KO.
- Supplemental Figure 6: Time course of renal KIM-1 expression in *Bpgm*-KO.
- Supplemental Figure 7: *Bpgm*-KO leads to a change in gene expression pattern of key factors involved in ROS metabolism, inflammation and fibrosis.
- Supplemental Figure 8: *Bpgm*-KO leads to up-regulation of alpha-smooth muscle actin (α-SMA), indicative of cellular de-differentiation.
- Supplemental Figure 9: KIM-1 positive proximal tubules are in close proximity to distal tubules.
- Supplemental Figure 10: Immunofluorescence staining of kidney injury marker KIM-1 along with immune cell markers following 4 days of *Bpgm*-KO.
- Supplemental Figure 11: Verification of target specificity of the anti-BPGM antibody used in this study.

- Reaction kinetics used for modeling of glucose metabolism shown in Figure 5A.

Supplemental Table 1: ON-TARGETplus SMARTpool siRNA Horizon Discovery Target Sequences.

**ON-TARGETplus SMARTpool siRNA**	**Target Sequences**
ON-TARGETplus Mouse BPGM siRNA, Horizon Discovery, Cat. # L-058581-01-0005	GAUCAGAGGCGCAUCGAAU
	GAAAGGAUUGCUCCGGAAA
	AGACAUAGUACUUACGAUA
	AAAGCAAGUCCUCGAUAUU
ON-TARGETplus Non-targeting Pool, Horizon Discovery, Cat. # D-001810-10-20	UGGUUUACAUGUCGACUAA
	UGGUUUACAUGUUGUGUGA
	UGGUUUACAUGUUUUCUGA
	UGGUUUACAUGUUUUCCUA

Supplemental Table 2: List of primers used for qPCR.

target gene (mouse)	primer sequences (5´ to 3´)
Actb	Forward: 5'-CTGTCGAGTCGCGTCCACC
	Reverse: 5'-GGCCTCGTCACCCACATAGG
Bpgm	Forward: 5'-TGTGCGATGTGCCCTTGGAT
	Reverse: 5'-AACGGCACGCAGGTTCTCAT
Ccl2	Forward: 5'-GCAGTTAACGCCCCACTCAC
	Reverse: 5'-CCATTCCTTCTTGGGGTCAGC
Tgfb1	Forward: 5'-CGGAGAGCCCTGGATACCAA
	Reverse: 5'-TTGCAGGAGCGCACAATCAT
Tnf-α	Forward: 5'-TGTAGCCCACGTCGTAGCAAA
	Reverse: 5'-ACGGCAGAGAGGAGGTTGAC
Icam1	Forward: 5'-CCATCCCAAAGCTCGACACC
	Reverse: 5'-CTCCCTTCCGAGACCTCCAG
Nrf2	Forward: 5'-GTGGATCCGCCAGCTACTCC
	Reverse: 5'-GGGATATCCAGGGCAAGCGA
Keap1	Forward: 5'-CATGGCTCACAAAGTGGTGC
	Reverse: 5'-AAGAACTCCTCCTGCTTGGC
Hmox-1	Forward: 5'-AACATCGACAGCCCCACCAA
	Reverse: 5'-GCTAGCAGGCCTCTGACGAA

Supplemental Table 3: List of antibodies used.

**Antibody target**	**Host**	**Supplier/ RRID**
primary antibodies		
Aquaporin-2	polyclonal rat	#20102rs; BiCell Scientific, USA; RRID:AB_2910118
BPGM	polyclonal rabbit	#NBP1-86064, Novus Biologicals, USA; RRID:AB_11013664
Calbindin	monoclonal mouse	#C9848, Sigma-Aldrich, USA; RRID:AB_476894
CD3	monoclonal rabbit	#ab16669, abcam, UK; RRID:AB_443425
CASP3	Monoclonal mouse	#AM08377PU-N, Acris Antibodies, USA; RRID:AB_2035249
F4/80	monoclonal rat	#MCA497GA, Bio-Rad Laboratories, USA; RRID:AB_323806
HO-1	monoclonal rabbit	#ab68477, abcam, UK; RRID:AB_11156457
KIM-1	polyclonal goat	#AF1817, R and D Systems, USA; RRID:AB_2116446
LY-6G	polyclonal rat	#127601, BioLegend, USA; RRID:AB_1089180
Megalin	monoclonal mouse	#ab184676, abcam, UK; RRID:AB_2910117
NCC	polyclonal rabbit	#AB3553, Millipore, USA; RRID:AB_571116
NF-kB p65	monoclonal rabbit	#D14E12, Cell Signaling Technology, USA; RRID:AB_10859369
NGAL	polyclonal goat	#AF1857, R and D Systems, USA; RRID:AB_355022
NKCC2	polyclonal rabbit	#LS-C313275, Lifespan Biosciences, USA; RRID:AB_2910114
NKCC2	polyclonal goat	#ab240542, abcam, UK; RRID:AB_2910116
TUBB2B	polyclonal rabbit	#TA337744, OriGene Technologies, USA; RRID:AB_2910113
Vimentin	monoclonal rabbit	#ab92547, abcam, UK; RRID:AB_10562134
α-SMA	polyclonal rabbit	#ab15734, abcam, UK; RRID:AB_443242
secondary antibodies		
anti-goat Alexa Fluor 488	donkey	#705-545-147; Jackson Immuno Research Labs; RRID:AB_2336933
anti-goat Alexa Fluor 594	donkey	#705-585-147, Jackson Immuno Research Lab; RRID:AB_2340433
anti-rabbit-HRP	goat	#sc-2030, Santa Cruz Biotechnology, USA; RRID:AB_631747
anti-mouse Cy3	donkey	#715-165-150, Jackson Immuno Research Labs, RRID: AB_2340813
anti-rabbit Alexa Fluor 488	donkey	#711-545-152, Jackson Immuno Research Labs; RRID:AB_2313584
anti-rabbit Cy3	goat	#111-165-003, Jackson Immuno Research Labs, RRID: AB_2338000
anti-rat Cy3	donkey	#712-165-150, Jackson Immuno Research Labs, RRID:AB_2340666

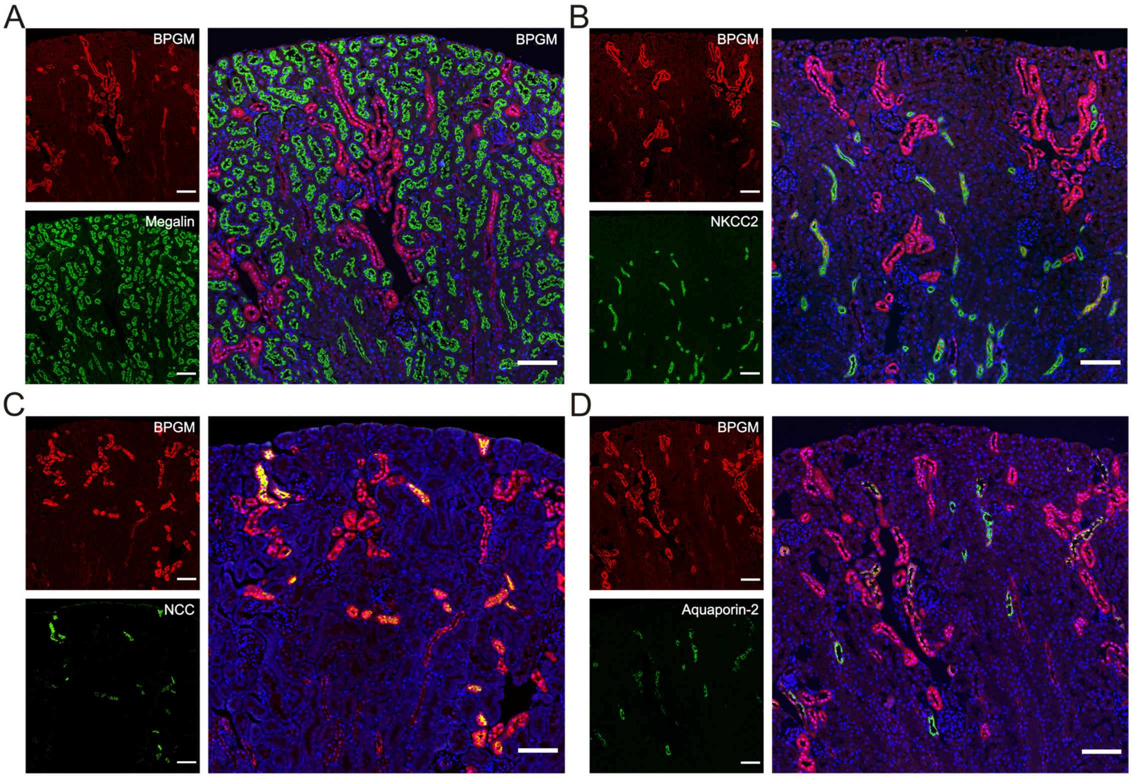

Supplemental Figure 1: BPGM localization in the kidney. (**A-D**) Immunofluorescence staining of mouse kidney section showing co-localization of BPGM (red) with marker proteins (green): (**A**) Megalin for PT (proximal tubules), (**B**) NKCC2 for TAL (thick ascending limb), (**C**) NCC for DCT (distal convoluted tubule), and (**D**) Aquaporin-2 for collecting duct. No BPGM expression was found in proximal tubules. Scale bar: 100 µm. The figure completes data shown in Figure 1B.

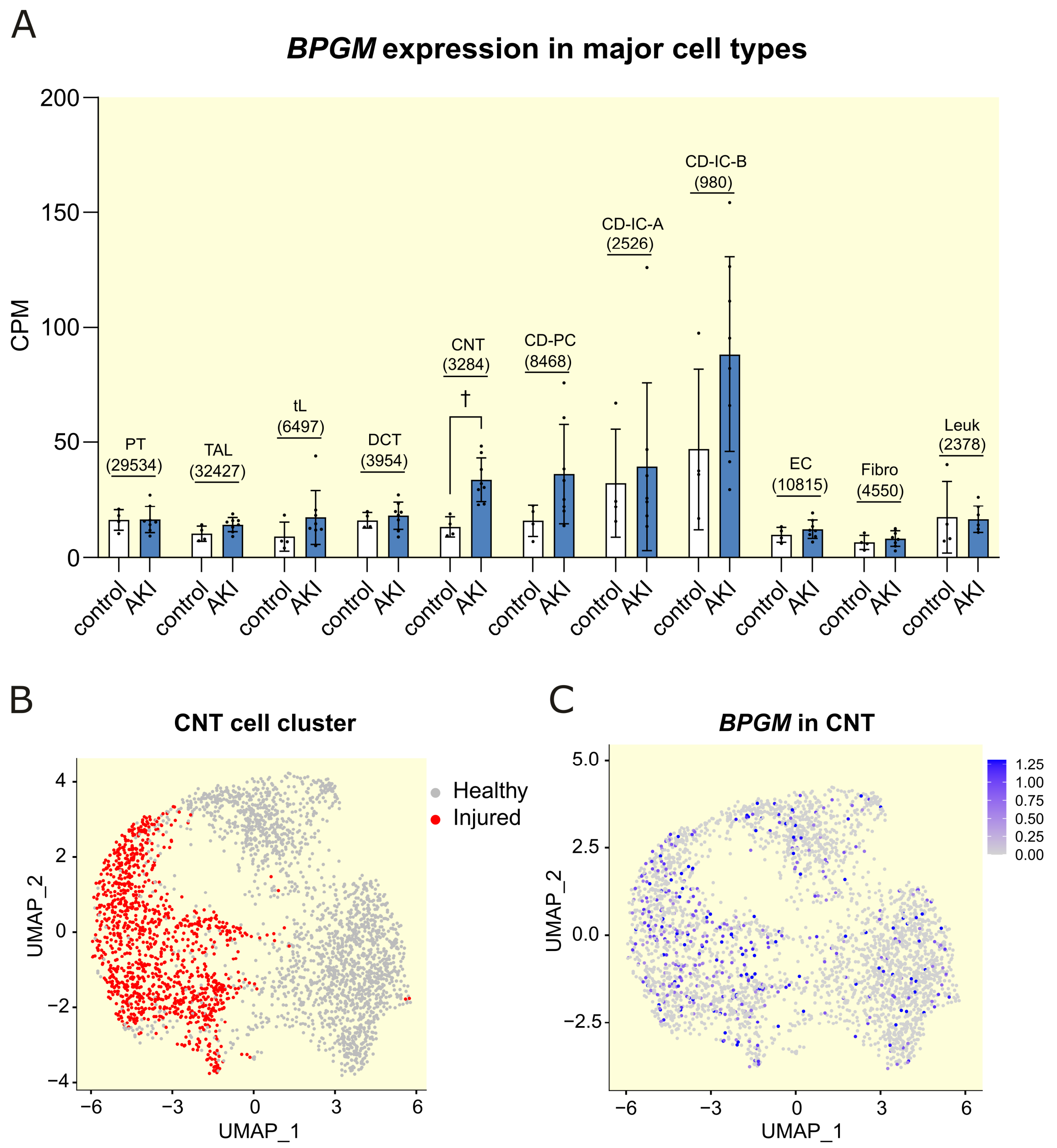

Supplemental Figure 2: Single-cell transcriptomic data from human AKI kidney biopsies. (**A-C**) Single-cell transcriptomic data from human kidney biopsies as described in Hinze *et al*. (12) (**A**) Expression values as counts per million (CPM) of *BPGM* mRNA in podocytes (Podo), proximal tubules (PT), thin limb of loop of Henle (tL), thick ascending limb (TAL), distal tubules (DCT), connecting tubules (CNT), collecting duct principal cells (CD-PC), CD intercalated cells type A (CD-IC-A) and type B (CD-IC-B), endothelial cells (EC), leukocytes (Leuko) and fibroblasts (Fibro). Cell numbers of clusters are parenthesized. †$p < 0.01$. (**B**) Uniform manifold approximation and projection (UMAP) of the sub-clustering for CNT. Cells from AKI-associated potentially injured CNT clusters are highlighted in red. (**C**) Feature plot of *BPGM* expression on CNT cells from AKI samples and controls. Expression values are log-normalized. Enrichment analysis indicates that *BPGM* is strongly enriched in injured cells ($p < 1e-20$).

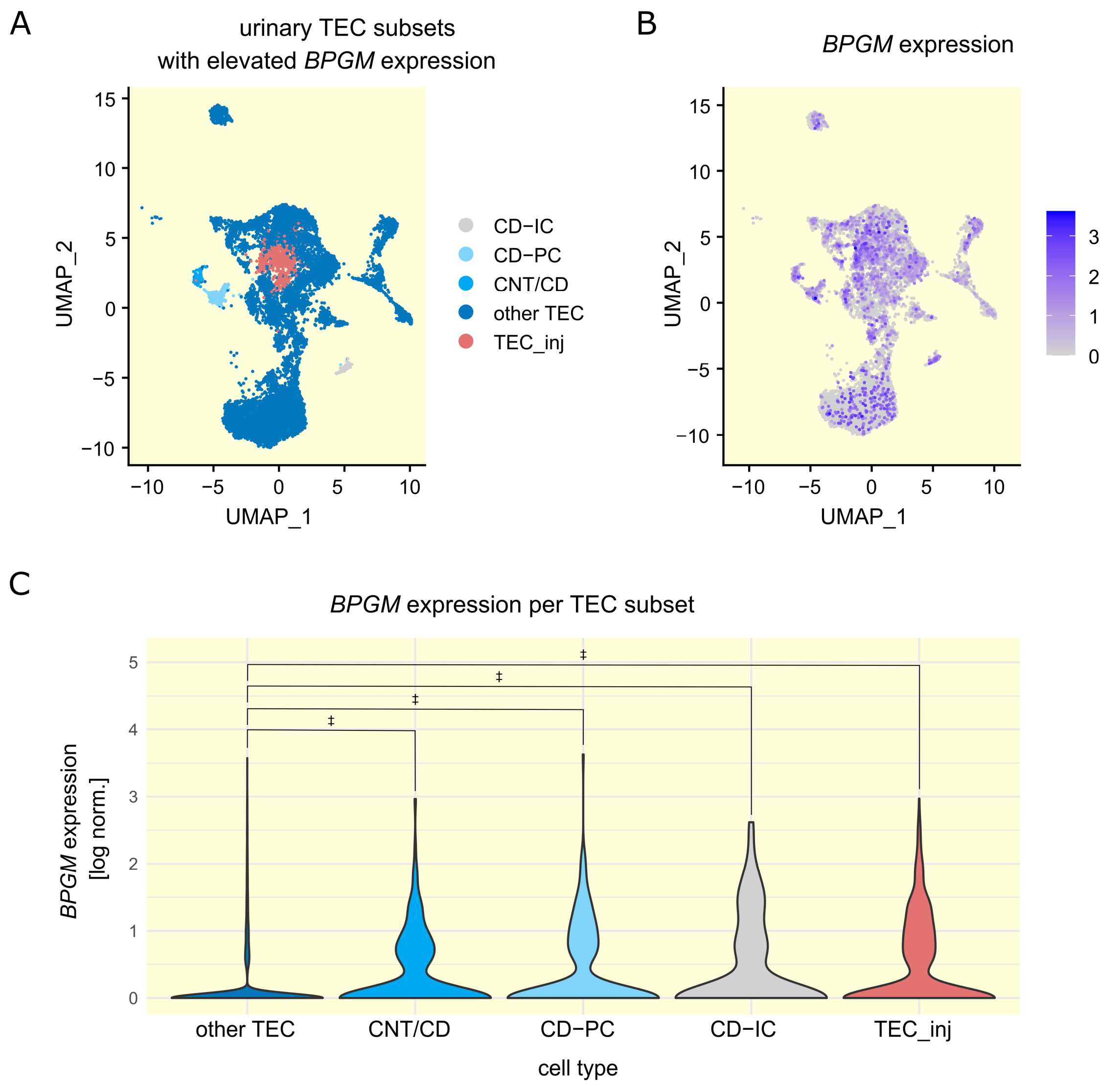

Supplemental Figure 3: Expression of *BPGM* in urine derived human kidney tubular epithelial cells following AKI. UMAP of 12,853 scRNAseq urine transcriptomes of tubular epithelial cells (TEC) from 32 individuals with AKI as described in Klocke *et al*. (13) (**A**) Depiction of TEC subsets with elevated *BPGM* expression in UMAP: *BPGM* expression is elevated in distal parts of the nephron, mostly connecting tubule (CNT), collecting duct principal cells (CD-PC) and intercalated cells (CD-IC) but also in a subset of injured TEC (TEC_inj). For full annotation of TEC subsets see Klocke *et al*. (13) (**B**) The magnitude of log-normalized expression of *BPGM* is depicted by colour code (grey to purple). (**C**) Violin plot of *BPGM* expression in each TEC subset. ‡$p < 0.001$. CNT/CD p-value < 3.484e-15, CD-PC p-value < 2.2e-16, CD-IC p-value < 3.641e-16, TEC_inj p-value < 2.2e-16.

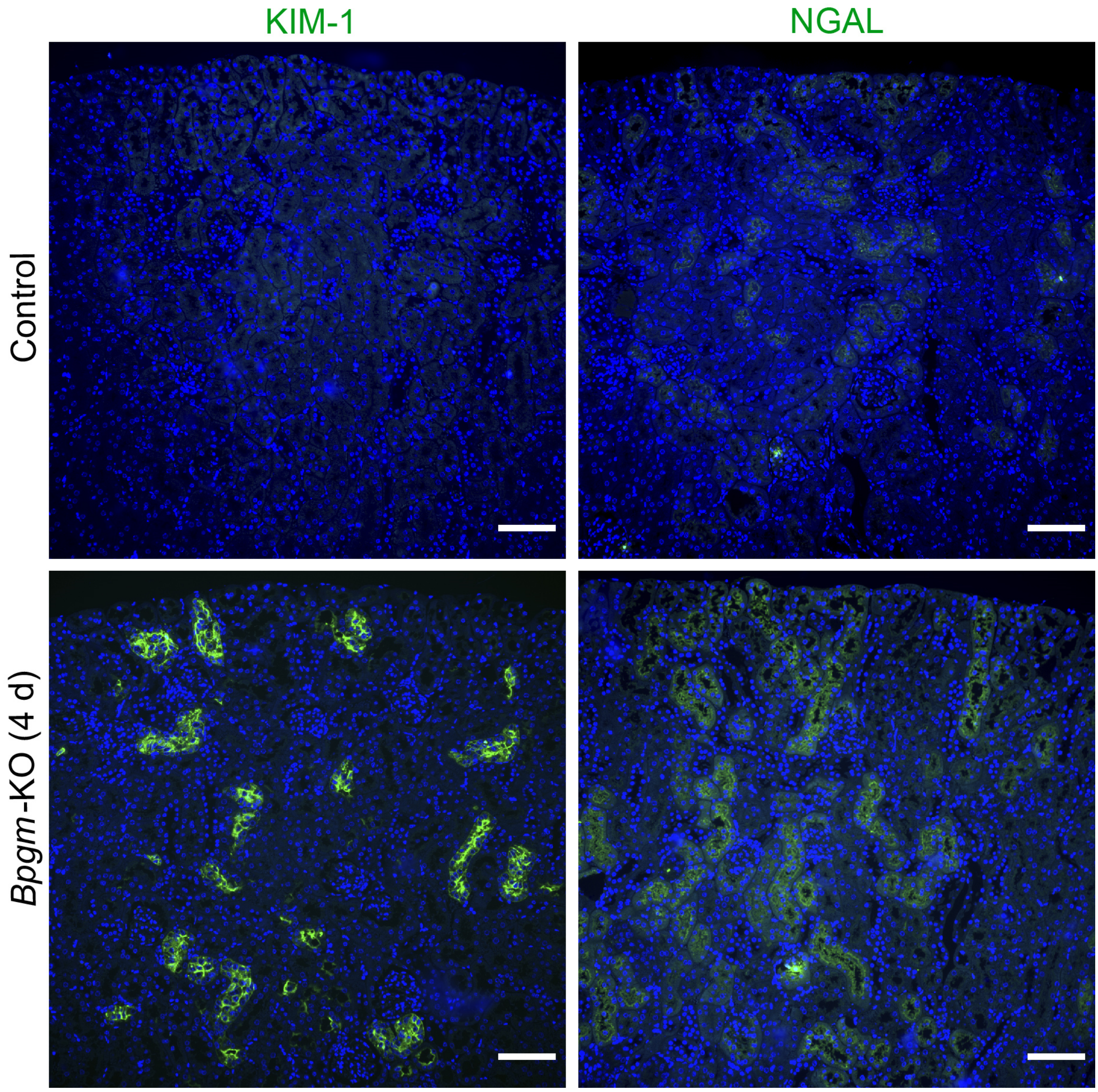

Supplemental Figure 4: Immunofluorescence staining of kidney injury markers KIM-1 and NGAL following 4 days of *Bpgm*-KO. Immunofluorescence staining of kidneys for injury markers KIM-1 and NGAL in control and *Bpgm*-KO mice 4 days after knockout induction. Control mice received doxycycline but were negative for *Cre* expression. KIM-1 and NGAL can only detected in Bpgm-KO animals. Scale bars: 100 µm.

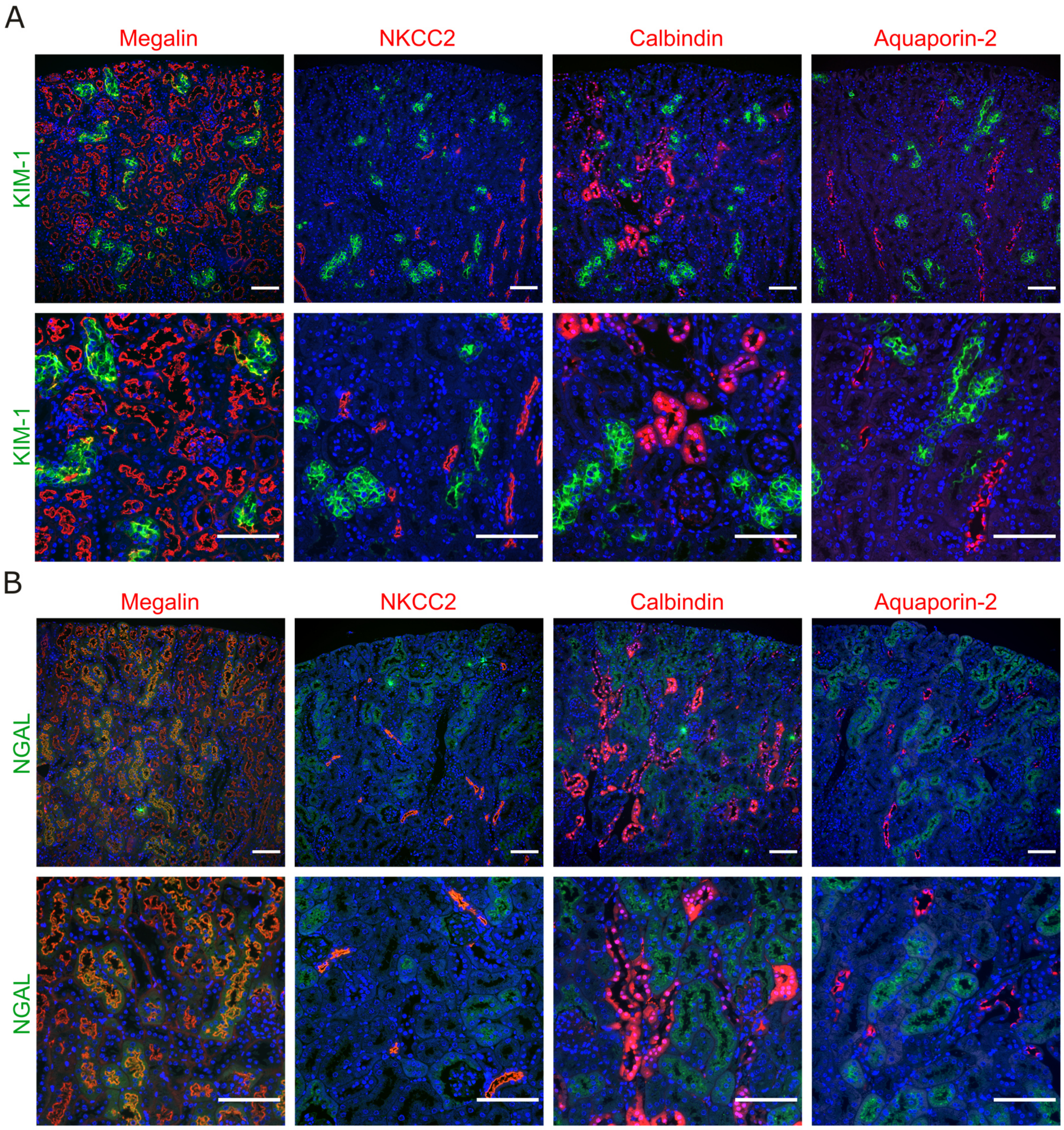

Supplemental Figure 5: Tubular localization of kidney injury markers KIM-1 and NGAL following 4 days of *Bpgm*-KO. A: Immunofluorescence double staining of KIM-1 (A) and NGAL (B) in green with marker proteins (red): Megalin for PT, NKCC2 for TAL, Calbindin for DCT and CNT, and Aquaporin-2 for collecting duct. (**A**) KIM-1 is co-localized with megalin, indicating damage of PT-cells. (**B**) NGAL is only co-localized with megalin, indicating damage of PT-cells. Scale bars: 100 µm.

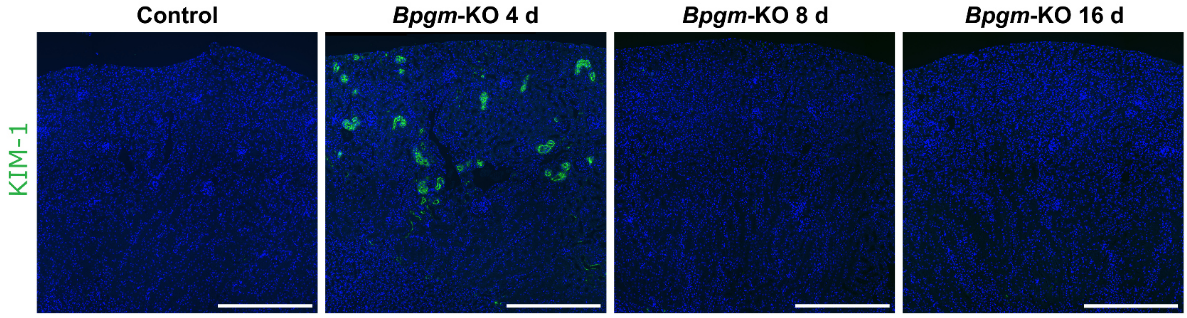

Supplemental Figure 6: Time course of renal KIM-1 expression in *Bpgm*-KO. Immunofluorescence staining for KIM-1 in kidneys of control and *Bpgm*-KO mice 4, 8 and 16 days after knockout induction. Control animals received doxycycline for the respective time point. KIM-1 positive tubules were observed only 4 days after *Bpgm*-KO. Scale bars: 500 µm.

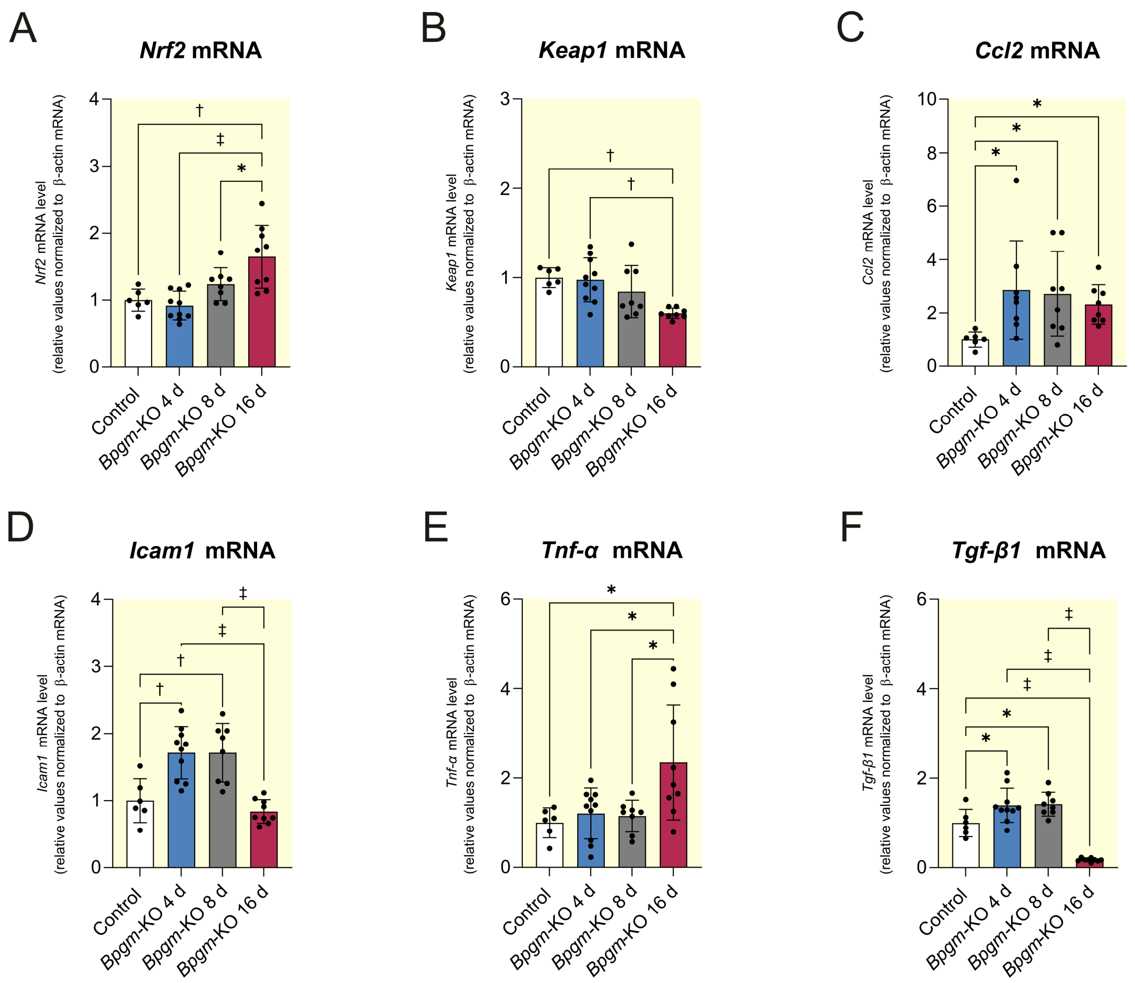

Supplemental Figure 7: *Bpgm*-KO leads to a marked change in gene expression pattern of key factors involved in ROS metabolism, inflammation and fibrosis. qPCR analyses of candidate genes. **A, B:** The transcription factor *Nrf2* (**A**) shows upregulation after 16 d of *Bpgm*-KO. In line, *Keap1*, an inhibitor of *Nrf2*, shows downregulation after 16 d (**B**). These data indicate adaptation to oxidative stress. **C-F**: Expression analysis of *Ccl2* (**C**), *Icam1* (**D**), *Tnf-α* (**E**) and *Tgf-β1* (**F**), genes that are associated with inflammation and fibrosis. Box-Plots show the median with lower and upper quartile as box. Whiskers show the minimum and maximum values. Dots represent single values. Statistical analysis was performed using One-Way Anova or Kuskal-Wallis test. *p<0.05; †p < 0.01; ‡p < 0.001.

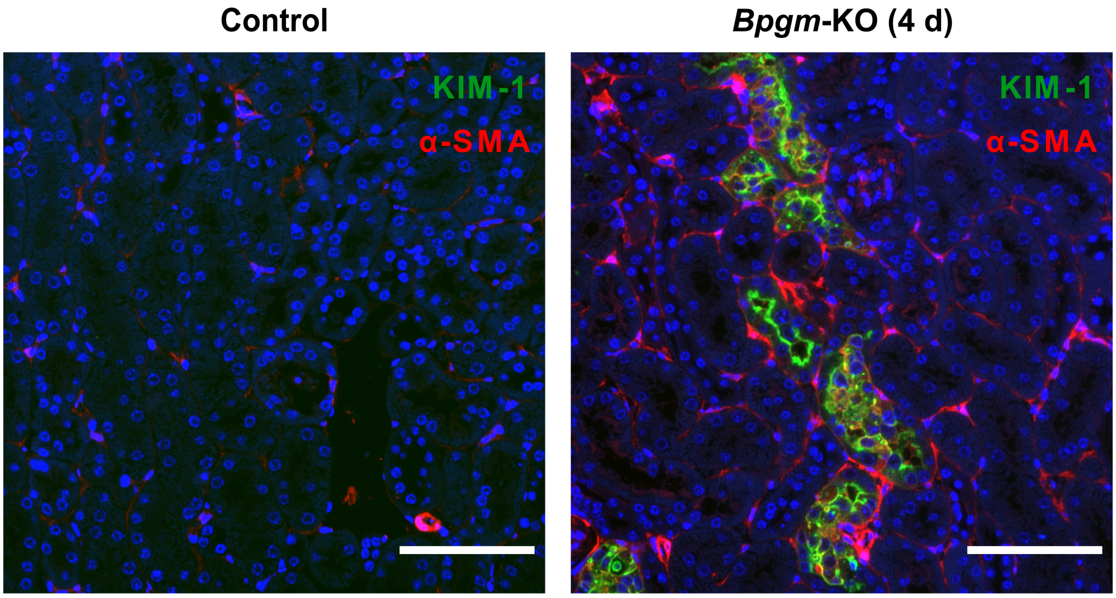

Supplemental Figure 8: *Bpgm*-KO leads to up-regulation of alpha-smooth muscle actin (α-SMA), indicative of cellular de-differentiation. Kidney sections were double stained for the proximal tubular injury marker KIM-1 (green) and the cellular *trans*-differentiation marker α-SMA (red). Expression of α-SMA is strongly enriched in KIM-1 positive cells following *Bpgm*-KO. Scale bars: 100 µm.

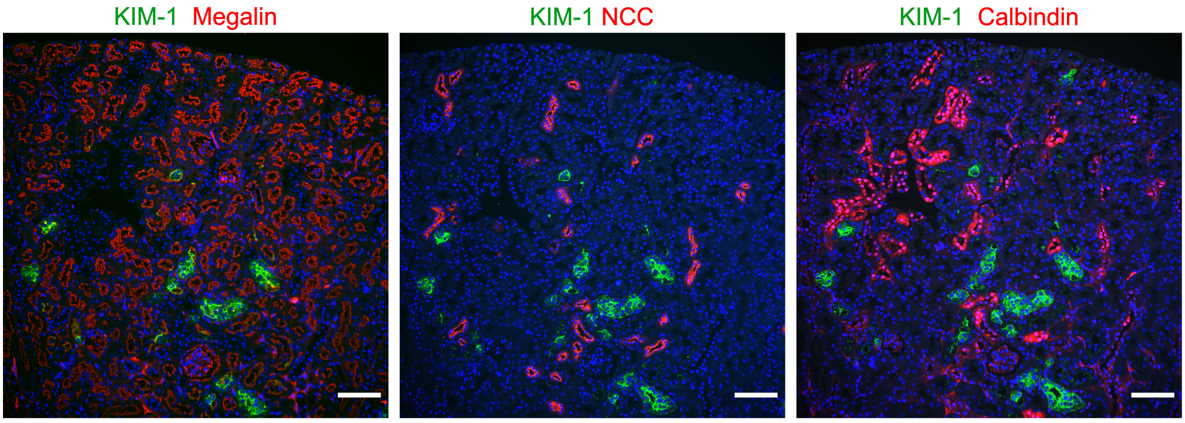

Supplemental Figure 9: KIM-1 positive proximal tubules are in close proximity to distal tubules. Immunofluorescence double staining of the kidney injury marker KIM-1 (green) and nephron segment markers (red): megalin for proximal tubules, NCC for distal convoluted tubules (DCT) and calbindin for the late part of the distal convolute tubule (DCT2) and connecting tubules (CNT). Data suggest that injured (KIM-1 positive) proximal tubules locate within one to three tubular profiles distance from the distal nephron segments affected by the *Bpgm* knockout. Scale bars 100 µm.

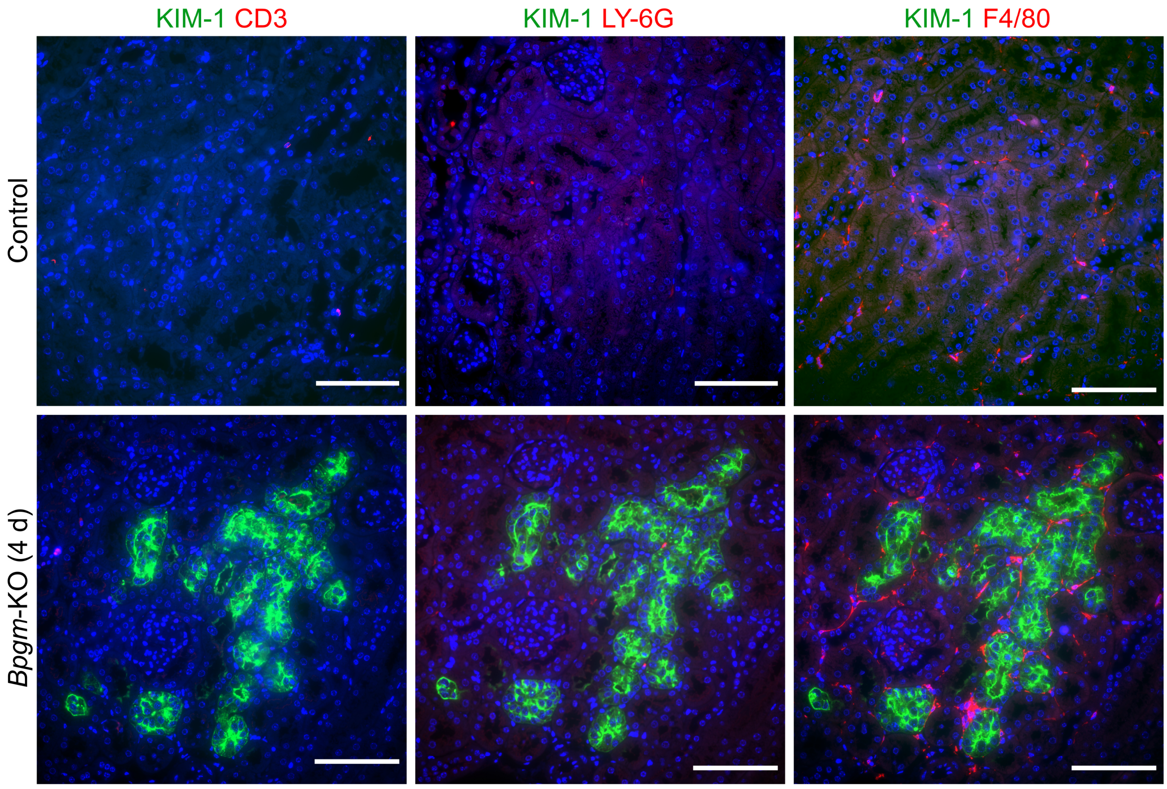

Supplemental Figure 10: Immunofluorescence staining of kidney injury marker KIM-1 along with immune cell markers following 4 days of *Bpgm*-KO. Immunofluorescence staining on parallel sections of kidney slices. The sections were double stained for the specific proximal tubular injury marker KIM-1 (green) and one of three immune cell markers (red), respectively: CD-3 for T-cells, Ly-6G for neutrophils and F4/80 for macrophages. Macrophages, but not neutrophils or T-cells surround the KIM-1 positive tubules. Scale bars: 100 µm.

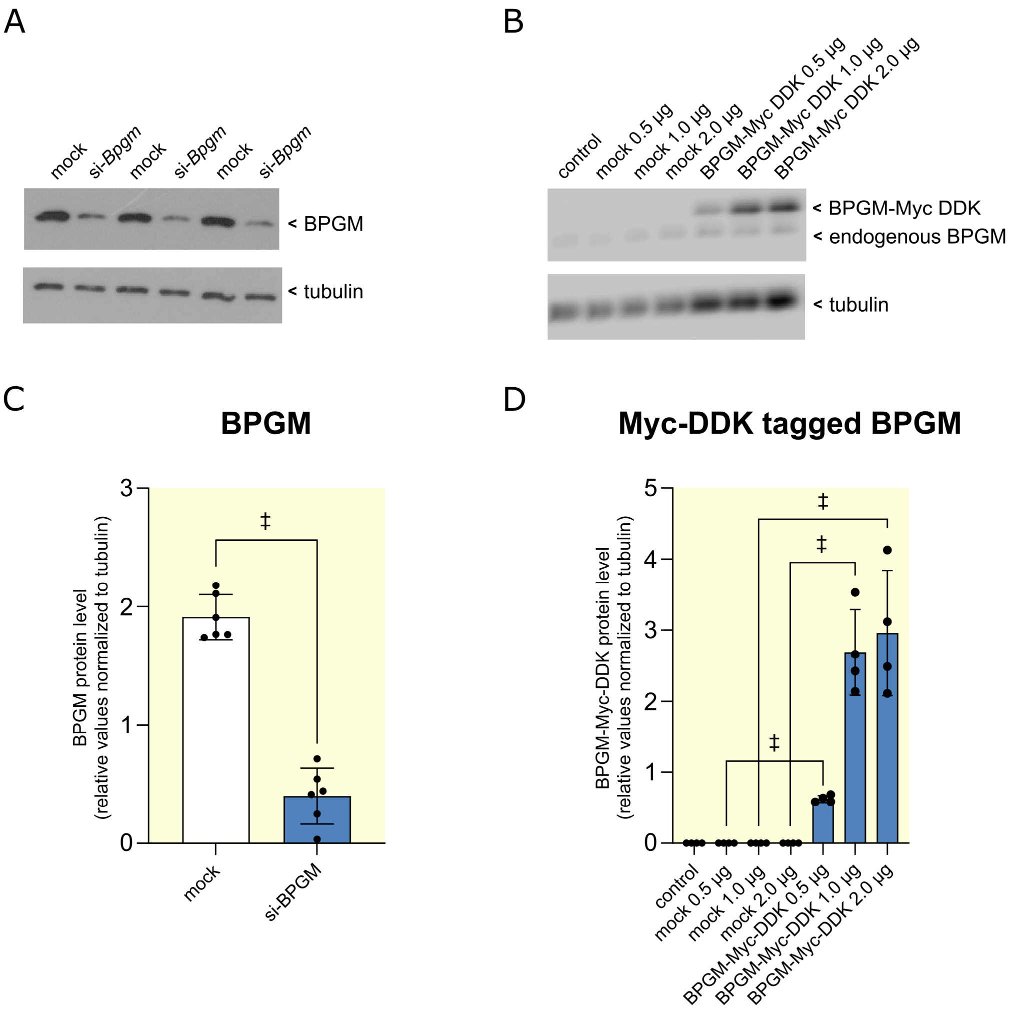

Supplemental Figure 11: Verification of target specificity of the anti-BPGM antibody used in this study. (**A, C**) Western blot analysis of siRNA-mediated knockdown of *Bpgm* (24 h). Knockdown lowered the BPGM protein signal intensity to about 20 % of the control value. N=6. (**B**, **D)** Western blot analysis of BPGM overexpression following transfection using a Myc-DDK tagged BPGM expression vector. The blot shows a dose dependent elevation of the signal intensity of the Myc-DDK tagged BPGM protein (detected at 30 kDa). Endogenous BPGM is detected at 27 kDa. Tubulin served as a loading control. N=4. Box-Plots show the median with lower and upper quartile as a box. Whiskers show the minimum and maximum values. Dots represent single values. Statistical analysis was performed using student's *t*-test. ‡p < 0.001.

REACTION KINETICS

Glycoly

ALD (Aldolase)

$$v_{ALD} = v_{max}^{ALD} \cdot \frac{Fru16P_2 - GAP \cdot DHAP/k_{eq}^{ALD}}{\left(1 + \frac{Fru16P_2}{k_m^{Fru16P_2} \cdot \left(1 + \frac{Bpg23}{K_{Bpg23}^i}\right)}\right) + \left(1 + \frac{GAP}{k_m^{GAP}}\right)\left(1 + \frac{DHAP}{k_m^{DHAP}}\right) - 1}$$

$v_{max}^{ALD} = 7.78 \cdot 10^8\, h^{-1}$

$K_{Bpg23}^i = 1.5$ [1]

$k_{eq}^{ALD} = 0.099$ mM [2]

$k_m^{Fru16P_2} = 0.004$ mM [3]

k_m^{GAP} =0.48 mM [4]

$K_m^{DHAP} = 0.38$ mM [4]

EN (Enolase)

$$v_{EN} = v_{max}^{EN} \cdot \frac{2PG - PEP/k_{eq}^{EN}}{1 + \frac{2PG}{k_m^{2PG}} + \frac{PEP}{k_m^{PEP}}}$$

$v_{max}^{EN} = 1.94 \cdot 10^{10} h^{-1}$

$k_{eq}^{EN} = 1.7$ [5]

$k_m^{2PG} = 0.14\, mM$ [6]

$k_m^{PEP} = 0.31\, mM$ [6]

FBP1 (Fructose-1,6-bisphosphatase)

$$v_{FBP1} = V_{max}^{FBP1} * \left((1 - \gamma^{FBP1}) * v_{FBP1}^{native} + \gamma^{FBP1} * v_{FBP1}^{phospho}\right)$$

$V_{max}^{FBP1} = 2.92 \cdot 10^4 mM \cdot h^{-1}$

$$v_{FBP1}^{native} = \frac{Fru16P_2}{Fru16P_2 + k_{m^{native}}^{Fru16P_2}} / \left(1 + \frac{Fru26P_2^n}{(k_i^{Fru26P_2})^n}\right) / \left(1 + \left(\frac{AMP}{K_i^{AMP}}\right)^{n_{AMP}}\right)$$

$k_{m^{native}}^{Fru16P_2} = 0.0029$mM [7]

$k_i^{Fru26P_2} = 0.00113$ mM [7]

$n = 1.26$ [7]

$n_{AMP} = 2.43$ [7]

$K_i^{AMP} = 0.023$ mM [7]

$$v_{FBP1}^{phospho} = \frac{Fru16P_2}{Fru16P_2 + k_{m^{phospho}}^{Fru16P_2}} / \left(1 + \frac{Fru26P_2^n}{(k_i^{Fru26P_2})^n}\right) / \left(1 + \left(\frac{AMP}{K_i^{AMP}}\right)^{n_{AMP}}\right)$$

$k_{m^{phospho}}^{Fru16P_2} = 0.0019$ mM [7]

$k_i^{Fru26P_2} = 0.00113$ mM [7]

$n = 1.26$ [7]

$K_i^{AMP} = 0.023$ mM [7]

$n_{AMP} = 2.43$ [7]

GAPDH (Glyceraldehyde 3-phosphate dehydrogenase)

$$v_{GAPDH} = v_{max}^{GAPDH} \cdot \frac{NAD^+ \cdot GAP \cdot P - 13P2G \cdot NADH / k_{eq}^{GAPDH}}{\left(1 + \frac{NAD^+}{k_m^{NAD^+}}\right) \cdot \left(1 + \frac{GAP}{k_m^{GAP}}\right) \cdot \left(1 + \frac{P}{k_m^P}\right) + \left(1 + \frac{NADH}{k_m^{NADH}}\right) \cdot \left(1 + \frac{13P2G}{k_m^{13P2G}}\right) - 1}$$

$v_{max}^{GAPDH} = 2.92 \cdot 10^8 h^{-1} \cdot mM^{-2}$

$k_{eq}^{GAPDH} = 10^{-4} mM^{-1}$ [8]

$k_m^{NAD^+} = 0.010$ mM [9]

$k_m^{GAP} = 0.035$ mM [9]

$k_m^P = 3.8$ mM [10]

$k_m^{NADH} = 0.006$ mM [10]

$k_m^{13P2G} = 0.01$ mM [9]

Hexokinase

$$v_{Hk} = V_{max}^{Hk} \cdot \frac{1}{1 + \frac{Bpg23}{K_{Bpg23}^{i}}} \frac{ATP}{ATP + K_m^{ATP} \cdot \left(1 + \frac{Glc6p}{K_{Glc6p}^{i-ATP}}\right)} \cdot \frac{Glc}{Glc + K_m^{Glc}\left(1 + \frac{Glc6p}{K_{Glc6p}^{i-Glc}}\right)}$$

$V_{max}^{Hk} = 90$

$K_{Bpg23}^{i} = 4$ [11]

$K_m^{Glc} = 0.42$ [12]

$K_m^{ATP} = 2.09$ [12]

$K_{Glc6p}^{i-ATP} = 0.19$ [12]

$K_{Glc6p}^{i-ATP} = 0.5$ [12]

Glucokinase

$$v_{GK} = V_{max}^{GK} \cdot \frac{ATP}{ATP + k_m^{ATP}} \cdot \frac{(Glc)^n}{(Glc)^n + (k_m^{Glc})^n}$$

$$V_{max}^{GK} = V_0^{GK} \cdot \frac{(Glc)^{n2}}{(Glc)^{n2} + (k_a^{Glc})^{n2}} \cdot \left(1 - f \cdot \frac{Fru6P}{Fru6P + k_i^{Fru6P}}\right)$$

$V_0^{GK} = 1.05 \cdot 10^4 mM\ h^{-1}$

$n = 1.5$ [13]

$K_m^{Glc} = 9\ mM$ [13]

$k_m^{ATP} = 0.55\ mM$ [14]

$k_i^{Fru6P} = 0.005\ mM$ [15]

$f = 0.75$ [15]

$n2 = 3.7$ [16]

$k_a^{Glc} = 15.9\ mM$ [16]

$GlcT_{ER}$ (Glucose transport to ER)

$$v_{GlcT_{ER}} = V_{max}^{GlcT_{ER}} * \frac{(Glc - Glc_{ER})}{1 + \frac{Glc}{k_m^{Glc}} + \frac{Glc_{ER}}{k_m^{Glc_{ER}}}}$$

$V_{max}^{Glc_{ER}} = 1.94 \cdot 10^{10}\ h^{-1}$

$k_m^{Glc_{ER}} = 1.37\ mM$ [17, 18]

$k_m^{Glc} = 1.22\ mM$ [17, 18]

GLUT2 (Glucose transporter 2)

$$v_{GLUT2} = V_{max}^{GLUT2} \cdot \frac{Glc_{ext} - Glc}{1 + \frac{Glc_{ext}}{k_m^{Glc_{ext}}} + \frac{Glc}{k_m^{Glc}}}$$

$k_m^{Glc} = 17.3\ mM$ [19]

$k_m^{Glc_{ext}} = 17.3\ mM$ [19]

$V_{max}^{GLUT2} = 9.09 \cdot 10^1\ h^{-1}$

$G6P_{ER}$ (Glucose-6-phosphate phosphatase in the ER) [20, 21]

$$v_{G6P_{ER}} = V_{max}^{G6P_{ER}} \cdot \frac{Glc6P_{ER}}{Glc6P_{ER} + k_m^{Glc6P_{ER}}}$$

$k_m^{Glc6P_{ER}} = 1.84$ mM [20]

$V_{max}^{G6P_{ER}} = 4.57 \cdot 10^2\ mM \cdot h^{-1}$

GPI (Glucose-6-phosphate isomerase)

$$v_{GPI} = V_{max}^{GPI} \cdot \frac{Glc6P - Fru6P/k_{eq}^{GPI}}{1 + \frac{Glc6P}{k_m^{Glc6P}} + \frac{Fru6P}{k_m^{Fru6P}}}$$

$V_{max}^{GPI} = 1.07 \cdot 10^9 h^{-1}$

$k_{eq}^{GPI} =$ 0.3 [22]

$k_m^{Glc6P} = 0.055\ mM$ [23]

$K_m^{Fru6P_{cyt}} = 0.12$ mM [23]

$G6PT_{ER}$ (Glucose-6-phosphate transport to ER)

$$v_{G6PT_{ER}} = V_{max}^{G6PT_{ER}} \cdot \frac{(Glc6P - Glc6P_{ER})}{1 + \frac{Glc6P}{k_m^{Glc6P}} + \frac{Glc6P_{ER}}{k_m^{Glc6P_{ER}}}}$$

$V_{max}^{G6PT_{ER}} = 1.94 \cdot 10^{10}\ h^{-1}$

$k_m^{Glc6P_{ER}} = 1.12\ mM$ [17]

$k_m^{Glc6P} = 1.12\ mM$ [17]

LacT (Lactate transporter)

$$v_{LacT} = v_{max}^{LacT} \cdot \frac{Lac_{ext} - Lac}{1 + \frac{Lac}{k_m^{Lac}} + \frac{Lac_{ext}}{k_m^{Lac_{ext}}}}$$

$v_{max}^{LacT} = 5.83 \cdot 10^2\ h^{-1}$

$k_m^{Lac} = 2.42\ mM$ [24]

$k_m^{Lac_{ext}} = 2.42\ mM$ [24]

LDH (Lactate dehydrogenase) [25, 26]

$$v_{LDH} = v_{max}^{LDH} * \frac{Pyr \cdot NADH - Lac \cdot NAD^+/k_{eq}^{LDH}}{\left(1 + \frac{NADH}{k_m^{NADH}}\right) \cdot \left(1 + \frac{Pyr}{k_m^{Pyr}}\right) + \left(1 + \frac{Lac}{k_m^{Lac}}\right) \cdot \left(1 + \frac{NAD^+}{k_m^{NAD^+}}\right) - 1}$$

$v_{max}^{LDH} = 1.56 \cdot 10^{11} h^{-1} \cdot mM^{-1}$

$k_{eq}^{LDH} = 9000$ [27]

$k_m^{NADH} = 0.015$ mM [26]

$k_m^{Pyr} = 0.15\ mM$ [26]

$k_m^{Lac} = 36\ mM$ [25]

$k_m^{NAD^+} = 0.11$ mM [26]

MalT (Malate transporter)

$$v_{MalT} = v_{max}^{MalT} \cdot \left(\frac{Mal_{mito} \cdot P - Mal \cdot P_{mito}}{\left(1 + \frac{Mal_{mito}}{K_m^{Mal_{mito}}}\right) \cdot \left(1 + \frac{P}{K_m^P}\right) + \left(1 + \frac{Mal}{K_m^{Mal}}\right) \cdot \left(1 + \frac{P_{mito}}{K_m^{P_{mito}}}\right) - 1} \right)$$

$v_{max}^{MalT} = 1.94 \cdot 10^3 h^{-1} \cdot mM^{-1}$

k_m^P = 1.41 mM [28]

$k_m^{Mal_{mito}}$ = 0.49 mM [28]

$k_m^{P_{mito}}$ = 1.41 mM [28]

k_m^{Mal} = 0.49 mM [28]

MDH (Malate dehydrogenase)

$$v_{MDH} = v_{max}^{MDH} \cdot \frac{Mal \cdot NAD^+ - OA \cdot NADH / k_{eq}^{MDH}}{\left(1 + \frac{Mal}{k_m^{Mal}}\right) \cdot \left(1 + \frac{NAD^+}{k_m^{NAD^+}}\right) + \left(1 + \frac{OA}{k_m^{OA}}\right) \cdot \left(1 + \frac{NADH}{k_m^{NADH}}\right) - 1}$$

$v_{max}^{MDH} = 1.94 \cdot 10^9 h^{-1} \cdot mM^{-1}$

$k_{eq}^{MDH} = 3 \cdot 10^{-5}$ [29]

$k_m^{Mal} = 1.1\, mM$ [30]

$k_m^{NAD^+} = 0.114\, mM$ [30]

$k_m^{OA} = 0.088\, mM$ [30]

$k_m^{NADH} = 0.026\, mM$ [30]

MDH$_{mito}$ (Mitochondrial malate dehydrogenase)

$$v_{MDH_{mito}} = V_{max}^{MDH_{mito}} \cdot \left(\frac{Mal_{mito} \cdot NAD_{mito}^+ - {}^1\!/_{K_{eq}^{MDH_{mito}}} \cdot OA_{mito} \cdot NADH_{mito}}{\left(1 + \frac{Mal_{mito}}{K_m^{Mal_{mito}}}\right) \cdot \left(1 + \frac{NAD_{mito}}{K_m^{NAD_{mito}}}\right) + \left(1 + \frac{OA_{mito}}{K_m^{OA_{mito}}}\right) \cdot \left(1 + \frac{NADH_{mito}}{K_m^{NADH_{mito}}}\right) - 1} \right)$$

$V_{max}^{MDH_{mito}} = 6.80 \cdot 10^{11} h^{-1} \cdot mM^{-1}$

$K_{eq}^{MDH_{mito}} = 3.1 \cdot 10^{-5}$ (pH 7.5) [29]

$K_m^{Mal_{mito}}$ = 0.33 mM [31]

$K_m^{NAD_{mito}}$ = 0.06 mM [32]

$K_m^{OA_{mito}}$ = 0.017 mM [32]

$K_m^{NADH_{mito}}$ =0.044 mM [32]

PC (Pyruvate carboxylase) [33, 34]

$$v_{PC} = v_{max}^{PC} \cdot \frac{ATP_{mito} \cdot Pyr_{mito} \cdot CO_{2_{mito}} - OA_{mito} \cdot ADP_{mito} \cdot P_{mito}/k_{eq}^{PC}}{\left(ATP_{mito} + k_m^{ATP_{mito}}\right) \cdot \left(Pyr_{mito} + k_m^{Pyr_{mito}}\right) \cdot \left(CO_{2_{mito}} + k_m^{CO_{2mito}}\right)}$$

$v_{max}^{PC} = 3.59 \cdot 10^3 mM \cdot h^{-1}$

$k_m^{ATP_{mito}}$ = 0.14 mM [33]

$k_m^{Pyr_{mito}}$ = 0.33 mM [33]

$k_m^{CO_{2mito}}$ = 4.2 mM [33]

$k_{eq}^{PC} =$ 6.55 [34]

PEPCK (Phosphoenolpyruvate carboxykinase)

$$v_{PEPCK} = v_{max}^{PEPCK} \cdot \frac{OA \cdot GTP - PEP \cdot GDP * CO_2/k_{eq}^{PEPCK}}{\left(1 + \frac{OA}{k_m^{OA}}\right) \cdot \left(1 + \frac{GTP}{k_m^{GTP}}\right) + \left(1 + \frac{PEP}{k_m^{PEP}}\right) \cdot \left(1 + \frac{GDP}{k_m^{GDP}}\right) \cdot \left(1 + \frac{CO_2}{k_m^{CO_2}}\right) - 1}$$

$v_{max}^{PEPCK} = 5.11 \cdot 10^5 h^{-1} \cdot mM^{-1}$

k_{eq}^{PEPCK} =110 mM [35]

$k_m^{OA} =$ 0.024 mM [36]

k_m^{GTP} =0.021 mM [37]

k_m^{PEP} = 0.4 mM [38]

k_m^{GDP} = 0.02 mM [39]

$k_m^{CO_2}$ = 1.194 mM [40]

PEPCK$_{mito}$ (Mitochondrial phosphoenolpyruvate carboxykinase)

$v_{PEPCK_{mito}}$

$= v_{max}^{PEPCK_{mito}}$

$$\cdot \frac{OA_{mito} \cdot GTP_{mito} - PEP_{mito} \cdot GDP_{mito} \cdot CO_{2_{mito}} / k_{eq}^{PEPCK_{mito}}}{\left(1 + \frac{OA_{mito}}{k_{m^{mito}}^{OA}}\right) \cdot \left(1 + \frac{GTP_{mito}}{k_{m^{mito}}^{GTP}}\right) + \left(1 + \frac{PEP_{mito}}{k_m^{PEP_{mito}}}\right) \cdot \left(1 + \frac{GDP_{mito}}{k_m^{GDP_{mito}}}\right) \cdot \left(1 + \frac{CO_{2_{mito}}}{k_m^{CO_{2_{mito}}}}\right) - 1}$$

$v_{max}^{PEPCK_{mito}} = 1.0 \cdot 10^6 h^{-1} \cdot mM^{-1}$

$k_{eq}^{PEPCK_{mito}}$ =160 mM [35]

$k_m^{OA_{mito}} = 0.0085$ mM [41]

$k_m^{GTP_{mito}} = 0.022$ mM [39]

$k_m^{PEP_{mito}} = 0.4\ mM$ [38]

$k_m^{GDP_{mito}} = 0.02$ mM [39]

$k_m^{CO_{2_{mito}}} = 1.06$ mM [42]

PEPT (Phosphoenolpyruvate transporter)

$$v_{PEPT} = v_{max}^{PEPT} \cdot \frac{PEP_{mito} - PEP/k_{eq}^{PEPT}}{1 + \frac{PEP}{k_m^{PEP}} + \frac{PEP_{mito}}{k_m^{PEP_{mito}}}}$$

$v_{max}^{PEPT} = 1.94 \cdot 10^5\ h^{-1}$

$k_{eq}^{PEPT} = \exp\left(-\frac{Vmm \cdot F}{R \cdot T}\right)$

$k_m^{PEP} = 0.1\ mM$ [43]

$k_m^{PEP_{mito}} = 0.1\ mM$ [43]

PFK1 (Phosphofructokinase 1)

$$v_{PFK1} = v_{max}^{PFK1} \cdot \frac{1}{1 + \frac{Bpg23_{cyt}}{K_{Bpg23}^{i}}} \frac{ATP}{ATP + K_m^{ATP}} \cdot \left(1 - \frac{ATP^{n_i}}{ATP^{n_i} + \left(K_i^{ATP}\right)^{n_i}}\right)$$

$$\cdot \frac{(Fru6P)^{n_{Fru6P}}}{(Fru6P)^{n_{Fru6P}} + (k_m^{Fru6P})^{n_{Fru6P}}}$$

$v_{max}^{PFK1} = 7.68 \cdot 10^4 mM \cdot h^{-1}$

$K_{Bpg23}^{i} = 1$ [11]

$K_m^{ATP} = K_0^{ATP} \cdot \left(1 - \frac{Fru26P_2}{Fru26P_2 + K_a^{Fru26P_2}}\right)$

$K_0^{ATP} = 0.2$ mM [44, 45]

$K_a^{Fru26P_2} = 0.0027$ mM [44, 45]

$K_i^{ATP} = K_{i0}^{ATP} \cdot \left(1 + f_{Fru26P_2} \frac{Fru26P_2}{Fru26P_2 + K_{a2}^{Fru26P_2}}\right)$

$K_{i0}^{ATP} = 0.7$ mM [44, 45]

$f_{Fru26P_2} = 9$ [44, 45]

$K_{a2}^{Fru26P_2} = 0.54\, mM$ [44, 45]

$n_i = 4$ [44, 45]

$$k_m^{Fru6P} = K_0^{Fru6p} \cdot \left(1 + \frac{ATP}{k_i^{ATP}}\right) \cdot \left(1 + \frac{Cit}{k_i^{Cit}}\right) \cdot \left(1 - f_{AMP} \frac{AMP^{n_{AMP}}}{AMP^{n_{AMP}} + (K_a^{AMP})^{n_{AMP}}}\right) \cdot \left(1 - f_P \frac{P}{P + K_a^P}\right) \cdot \left(1 - f_{Fru26P_2} \frac{{Fru26P_2}^{n_{Fru26P_2}}}{{Fru26P_2}^{n_{Fru26P_2}} + \left(K_a^{Fru26P_2}\right)^{n_{Fru26P_2}}}\right)$$

$K_0^{Fru6P} = 1.14$ mM [46]

$k_i^{ATP} = 0.6$ mM [46]

$k_i^{Cit} = 3.27$ mM [46]

$f_{AMP} = 0.77$ [46]

$K_a^{AMP} = 0.1$ mM [46]

$n_{AMP} = 1.84$ [46]

$f_P = 0.85$ [46]

$K_a^P = 0.69$ mM [46]

$f_{Fru26P_2} = 0.92$ [44]

$K_a^{Fru26P_2} = 0.0045\, mM$ [44]

$n_{Fru26P_2} = 1.2$ [44]

$$n^{Fru6P} = \left(n_0 + \frac{ATP^{n_{ATP}}}{ATP^{n_{ATP}} + \left(K_i^{ATP}\right)^{n_{ATP}}}\right) \cdot \left(1 - f_{AMP} \frac{AMP^{n_{AMP}}}{AMP^{n_{AMP}} + (K_a^{AMP})^{n_{AMP}}}\right)$$

$$\cdot \left(1 + f_{Cit} \frac{Cit^{n_{Cit}}}{Cit^{n_{Cit}} + \left(K_i^{Cit}\right)^{n_{Cit}}}\right) \cdot \left(1 - f_P \frac{P^{n_P}}{P^{n_P} + (K_a^P)^{n_P}}\right) \cdot$$

$$\cdot \left(1 - f_{Fru26P_2} \frac{{Fru26P_2}^{n_{Fru26bp}}}{Fru26P_2^{n_{Fru26P_2}} + \left(K_a^{Fru26P_2}\right)^{n_{Fru26P_2}}}\right)$$

$n_0 = 3.67$ [46]

$K_i^{ATP} = 0.13$ mM [46]

$n_{ATP} = 1.59$ [46]

$f_{AMP} = 0.4$ [46]

$K_a^{AMP} = 0.086$ mM [46]

$n_{AMP} = 2.22$ [46]

$f_{Cit} = 0.1$ [46]

$K_i^{Cit} = 0.18$ mM [46]

$n_{Cit} = 4$ [46]

$f_P = 0.28$ [46]

$K_a^P = 0.53$ mM [46]

$n_P = 4$ [46]

$f_{Fru26P_2} = 0.37$ [44]

$K_a^{Fru26P_2} = 0.0021$ mM [44]

$n^{Fru26P_2} = 4$ [44]

PFK2/FBP2 (Phosphofructokinase 2/Fructose-2,6-bisphosphatase)

$$v_{PFK2} = (1 - \gamma^{PFK2}) \cdot v_{PFK2}^{native} + \gamma^{PFK2} \cdot v_{PFK2}^{phospho}$$

$$v_{PFK2}^{native} = V_{\max}^{PFK2} \frac{Fru6P^n}{Fru6P^n + \left(k_{m^{native}}^{Fru6P}\right)^n} \cdot \frac{ATP}{ATP + k_m^{ATP}} \cdot \left(1 - n_0 \cdot \frac{PEP}{PEP + k_i^{PEP}}\right)$$

$V_{max}^{PFK2} = 1.51 \cdot 10^2 \ mM \cdot h^{-1}$

$k_{m^{native}}^{Fru6P} = 0.015$ mM [47]

$n = 1.3$ [47]

$k_m^{ATP} = 0.25\, mM$ [47]

$k_i^{PEP} = 0.25\, mM$ [45]

$n_0 = 0.85$ [45]

$$v_{PFK2}^{phospho} = V_{max}^{PFK2} \frac{Fru6P^n}{Fru6P^n + {k_{m^{phospho}}^{Fru6P}}^n} \cdot \frac{ATP}{ATP + k_m^{ATP}} \cdot \left(1 - n_0 \cdot \frac{PEP}{PEP + k_i^{PEP}}\right)$$

$k_{m^{phospho}}^{Fru6P} = 0.05$ mM [47]

$n = 2$ [47]

$k_m^{ATP} = 0.5\, mM$ [47]

$k_i^{PEP} = 0.25\, mM$ [45]

$n_0 = 0.85$ [45]

$$v_{FBP2} = V_{max}^{FBP2} * \left((1 - \gamma^{FBP2}) \cdot v_{FBP2}^{native} + \gamma^{FBP2} \cdot v_{FBP2}^{phospho}\right)$$

$V_{max}^{FBP2} = 5.49 \cdot 10^2\, mM \cdot h^{-1}$

$$v_{FBP2}^{native} = \frac{Fru26P_2}{Fru26P_2 + k_{m^{native}}^{Fru26P_2}} / \left(1 + \frac{Fru6P}{k_{i^{native}}^{Fru6P}}\right)$$

$k_{m^{native}}^{Fru26P_2} = 0.01\, mM$ [48]

$k_{i^{native}}^{Fru6P} = 0.0035$ mM [47]

$$v_{FBP2}^{phospho} = \frac{Fru26P_2}{Fru26P_2 + k_{m^{phospho}}^{Fru26P_2}} / \left(1 + \frac{Fru6P}{k_{i^{phospho}}^{Fru6P}}\right)$$

$k_{m^{phospho}}^{Fru26P_2} = 0.0005$ mM [47]

$k_{i^{phospho}}^{Fru6P} = 0.01\, mM$ [47]

PGK (Phosphoglycerate kinase)

$$v_{PGK} = v_{max}^{PGK} * \frac{ADP \cdot 13P2G - ATP \cdot 3PG / k_{eq}^{PGK}}{\left(1 + \frac{ADP}{k_m^{ADP}}\right) \cdot \left(1 + \frac{13P2G}{k_m^{13P2G}}\right) + \left(1 + \frac{ATP}{k_m^{ATP}}\right) \cdot \left(1 + \frac{3PG}{k_m^{3PG}}\right) - 1}$$

$v_{max}^{PGK} = 1.94 \cdot 10^{10} h^{-1} \cdot mM^{-1}$

$k_{eq}^{PGK} = 1830$ [49]

$k_m^{ADP} = 0.35\ mM$ [50]

$k_m^{13P2G} = 0.0022\ mM$ [50]

$k_m^{ATP} = 0.24\ mM$ [51]

$k_m^{3PG} = 1.65\ mM$ [51]

BPGP (Bisphosphoglycerate Phosphatase)

$$v_{BPGP} = V_{max}^{BPGP} \cdot \frac{BPG23_{cyt}}{BPG23_{cyt} + K_m^{BPG23_{cyt}}}$$

$K_m^{BPG23_{cyt}} = 0.0051$ [52]

$V_{max}^{BPGP} = 5.832\ mM \cdot h^{-1}$

BPGM (Bisphosphoglycerate Phosphatase)

$$v_{BPGM} = V_{max}^{BPGM} \cdot \frac{BPG13_{cyt}}{BPG213_{cyt} + K_m^{BPG13_{cyt}} \cdot \left(1 + \frac{BPG23_{cyt}}{K_i^{BPG13_{cyt}}}\right)}$$

$V_{max}^{BPGM} = 583.2\ mM \cdot h^{-1}$

$K_m^{BPG23_{cyt}} = 0.004$ [53]

$K_i^{BPG13_{cyt}} = 0.11$ [53]

PGM (Phosphoglycerate mutase)

$$v_{PGM} = v_{max}^{PGM} \cdot \frac{3PG - 2PG/k_{eq}^{PGM}}{1 + \frac{3PG}{k_m^{3PG}} + \frac{2PG}{K_m^{2PG}}}$$

$v_{max}^{PGM} = 1.94 \cdot 10^{10}\ h^{-1}$

$k_{eq}^{PGM} = 0.096$ [54]

$k_m^{3PG} = 0.52\ mM$ [55]

$K_m^{2PG} = 0.24\ mM$ [55]

PK (Pyruvate kinase)

$$v_{PK} = v_{max}^{PK} \cdot \left((1-\gamma^{PK}) \cdot v_{PK}^{native} + \gamma^{PK} \cdot v_{PK}^{phospho}\right)$$

$$v_{PK}^{native} = \frac{PEP}{PEP + k_{m^{native}}^{PEP} \cdot \left(1 + \frac{ATP}{k_{i^{native}}^{ATP}}\right) \cdot \left(1 - \frac{Fru16P_2}{Fru16P_2 + k_{a^{native}}^{Fru16P_2}}\right)} \cdot \frac{ADP}{ADP + k_m^{ADP}}$$

$v_{max}^{PK} = 1.28 \cdot 10^4\ mM \cdot h^{-1}$

$k_{m^{native}}^{PEP}$ =0.13 mM [56]

$k_{i^{native}}^{ATP}$ =1 mM [56]

$k_{a^{native}}^{Fru16P_2}$ =0.0078 mM [57]

k_m^{ADP} = 0.25 mM [58]

$$v_{PK}^{phospho} = \frac{PEP^n}{PEP^n + \left(k_{m^{phospho}}^{PEP} \cdot \left(1 + \frac{ATP}{k_{i^{phospho}}^{ATP}}\right) \cdot \left(1 - \frac{Fru16P_2}{Fru16P_2 + k_{a^{phospho}}^{Fru16P_2}}\right)\right)^n} \cdot \frac{ADP}{ADP + k_m^{ADP}}$$

$k_{m^{phospho}}^{PEP}$ = 5.8 mM [56]

n = 2.9 [56]

$k_{a^{phospho}}^{Fru16P_2}$ = 0.0095 mM [57]

$k_{i^{phospho}}^{ATP}$ = 0.32 mM [59]

k_m^{ADP} = 0.33 mM [58]

PyrMalT (Pyruvate/malate antiporter)

$$v_{PyrMalT} = v_{max}^{PyrMalT} \cdot \left(\frac{Mal_{mito} \cdot Pyr - Mal \cdot Pyr_{mito}}{\left(1 + \frac{Mal_{mito}}{K_m^{Mal_{mito}}}\right) \cdot \left(1 + \frac{Pyr}{K_m^{Pyr}}\right) + \left(1 + \frac{Mal}{K_m^{Mal}}\right) \cdot \left(1 + \frac{Pyr_{mito}}{K_m^{Pyr_{mito}}}\right) - 1}\right)$$

$v_{max}^{PyrMalT} = 1.94 \cdot 10^4\ h^{-1} \cdot mM^{-1}$

k_m^{Pyr} = 0.84 mM [60]

k_m^{Mal} = 0.7 mM [43]

$k_m^{Pyr_{mito}} = 0.84\ mM$ [60]

$k_m^{Mal_{mito}} = 0.7\ mM$ [43]

PyrT (Pyruvate transporter)

$$v_{PyrT} = v_{max}^{PyrT} \cdot \frac{Pyr \cdot H^+ - Pyr_{mito} \cdot H_{mito}^+}{1 + \frac{Pyr}{k_m^{Pyr}} + \frac{Pyr_{mito}}{k_m^{Pyr_{mito}}}}$$

$v_{max}^{PyrT} = 1.94 \cdot 10^8 h^{-1} \cdot mM^{-1}$

$k_m^{Pyr} = 0.15$ mM [61]

$k_m^{Pyr_{mito}} = 0.15$ mM [61]

TPI (Triosephosphate isomerase)

$$v_{TPI} = v_{max}^{TPI} \cdot \frac{DHAP - GAP/k_{eq}^{TPI}}{1 + \frac{DHAP}{k_m^{DHAP}} + \frac{GAP}{k_m^{GAP}}}$$

$v_{max}^{TPI} = 1.94 \cdot 10^8\ h^{-1}$

$k_{eq}^{TPI} = 0.04545$ [2]

$k_m^{DHAP} = 0.59$ mM [62]

$k_m^{GAP} = 0.415$ mM [62]

Polyol pathway

Aldose reductase

$$v_{aldr} = V_{max}^{aldr} \cdot \left(\frac{Glc_{cyt}}{Glc_{cyt} + K_m^{glc_{cyt}}}\right) \cdot \left(\frac{Nadh_{cyt}}{Nadh_{cyt} + K_m^{nadh_{cyt}}}\right)$$

$V_{max}^{aldr} = 2.16$

$K_m^{glc_{cyt}} = 155$ [63]

$K_m^{nadh_{cyt}} = 0.01$ [63]

Sorbital dehydrogenase

$$v_{sordh} = V_{max}^{sordh} \cdot \frac{\left(Fru_{cyt} \cdot Nadh_{cyt} - {}^{1}\!/_{K_{eq}^{sordh}} \cdot Sorb_{cyt} \cdot Nad_{cyt}\right)}{\left(1 + \frac{Fru_{cyt}}{K_m^{fru_{cyt}}}\right) \cdot \left(1 + \frac{Nadh_{cyt}}{K_m^{nadh_{cyt}}}\right) + \left(1 + \frac{Sorb_{cyt}}{K_m^{sorb_{cyt}}}\right) \cdot \left(1 + \frac{Nad_{cyt}}{K_m^{nad_{cyt}}}\right) - 1}$$

$V_{max}^{sordh} = 3.6$ [2]

$K_m^{fru_{cyt}} = 136$ [64]

$K_m^{nadh_{cyt}} = 0.067$ [64]

$K_m^{sorb_{cyt}} = 0.38$ [64]

$K_m^{nad_{cyt}} = 0.082$ [64]

Fructokinase

$$v_{fruk} = V_{max}^{fruk} \cdot \left(\frac{Fru_{cyt}}{Fru_{cyt} + K_m^{fru_{cyt}}}\right) \cdot \left(\frac{ATP_{cyt}}{ATP_{cyt} + K_m^{atp_{cyt}}}\right) \cdot \left(1 - \frac{ADP_{cyt}^{n}}{ADP_{cyt}^{n} + \left(K_i^{adp_{cyt}}\right)^{n}}\right)$$

$V_{max}^{fruk} = 3.6$

$K_m^{fru_{cyt}} = 0.4$ [38]

$K_m^{atp_{cyt}} = 1.5$ [38]

$n = 1.5$ [65]

$K_i^{adp_{cyt}} = 3.06$ [65]

Aldolase B

$$v_{aldB} = V_{max}^{aldB} \cdot \frac{\left(Fru1p_{cyt} - {}^{1}\!/_{K_{eq}^{aldB}} \cdot Gra_{cyt} \cdot Dhap_{cyt}\right)}{\left(1 + \frac{Fru1p_{cyt}}{K_m^{fru1p_{cyt}}}\right) + \left(1 + \frac{Gra_{cyt}}{K_m^{gra_{cyt}}}\right) \cdot \left(1 + \frac{Dhap_{cyt}}{K_m^{dhap_{cyt}}}\right) - 1}$$

$V_{max}^{aldB} = 3.6$

$K_{eq}^{aldB} = 0.0028$ [66]

$K_m^{fru1p_{cyt}} = 4.5$ [67]

$K_m^{gra_{cyt}} = 0.48$ [4]

$K_m^{dhap_{cyt}} = 0.016$ [68]

Triokinase

$$v_{triok} = V_{max}^{triok} \cdot \left(\frac{Gra_{cyt}}{Gra_{cyt} + K_m^{gra_{cyt}}} \right) \cdot \left(\frac{ATP_{cyt}}{ATP_{cyt} + K_m^{atp_{cyt}}} \right)$$

$V_{max}^{triok} = 180$

$K_m^{gra_{cyt}} = 0.03$ [69]

$K_m^{atp_{cyt}} = 0.2$ [69]

STOICHIOMETRIC MATRIX

$$\frac{d}{dt}DHAP = v_{ALD} - v_{TPI} + v_{aldB}$$

$$\frac{d}{dt}Fru_{cyt} = +v_{sordh} - v_{fruk}$$

$$\frac{d}{dt}Fru1p_{cyt} = +v_{fruk} - v_{aldB}$$

$$\frac{d}{dt}Fru16P_2 = v_{PFK1} - v_{FBP1} - v_{ALD}$$

$$\frac{d}{dt}Fru26P_2 = v_{PFK2} - v_{FBP2}$$

$$\frac{d}{dt}Fru6P = v_{GPI} - v_{PFK1} - v_{PFK2} + v_{FBP1} + v_{FBP2}$$

$$\frac{d}{dt}GAP = v_{ALD} + v_{TPI} - v_{GAPDH} + v_{triok}$$

$$\frac{d}{dt}Glc = v_{GLUT2} - v_{GK} + v_{GlcT_{ER}} - v_{aldr}$$

$$\frac{d}{dt}Glc_{ER} = v_{G6P_{ER}} - v_{GlcT_{ER}}$$

$$\frac{d}{dt}Glc1P = v_{GP} - v_{G1PI} - v_{UGT}$$

$$\frac{d}{dt}Glc6P = v_{GK} + v_{G6PT_{ER}} - v_{GPI} + v_{G1PI}$$

$$\frac{d}{dt}Glc6P_{ER} = -v_{G6P_{ER}} - v_{Glc6PT_{ER}}$$

$$\frac{d}{dt}Gra_{cyt} = +v_{aldB} - v_{triok}$$

$$\frac{d}{dt}Lac = v_{LacT} + v_{LDH}$$

$$\frac{d}{dt}Mal = v_{MalT} - v_{MDH} + v_{PyrMalT}$$

$$\frac{d}{dt}Mal_{mito} = -v_{MalT} - v_{MDH_{mito}} - v_{PyrMalT}$$

$$\frac{d}{dt}OA = v_{MDH} - v_{PEPCK}$$

$$\frac{d}{dt}OA_{mito} = v_{PC} - v_{PEPCK_{mito}} + v_{MDH\,mito}$$

$$\frac{d}{dt}PEP = v_{EN} - v_{PK} + v_{PEPCK} - v_{PEPT}$$

$$\frac{d}{dt}PEP_{mito} = v_{PEPCK_{mito}} + v_{PEPT}$$

$$\frac{d}{dt}13P2G = v_{GAPDH} - v_{PGK}$$

$$\frac{d}{dt}23P2G = v_{BPGM} - v_{BPGP}$$

$$\frac{d}{dt}2PG = v_{PGM} - v_{EN}$$

$$\frac{d}{dt}3PG = v_{PGK} - v_{PGM}$$

$$\frac{d}{dt}Pyr = v_{PK} - v_{LDH} - v_{PyrT} - v_{PyrMalT}$$

$$\frac{d}{dt}Pyr_{mito} = v_{PyrT} - v_{PC} + v_{PyrMalT}$$

$$\frac{d}{dt}Sorb_{cyt} = +v_{aldr} + v_{sordh}$$

REFERENCES KINETIC MODEL

1. Srivastava, S.K. and E. Beutler, *The effect of normal red cell constituents on the activities of red cell enzymes.* Arch Biochem Biophys, 1972. **148**(1): p. 249-55. DOI: 10.1016/0003-9861(72)90138-5.
2. Veech, R.L., et al., *Disequilibrium in the triose phosphate isomerase system in rat liver.* Biochem J, 1969. **115**(4): p. 837-42.
3. Ikehara, Y., H. Endo, and Y. Okada, *The identity of the aldolases isolated from rat muscle and primary hepatoma.* Arch Biochem Biophys, 1970. **136**(2): p. 491-7. DOI: 0003-9861(70)90220-1 [pii].
4. Malay, A.D., S.L. Procious, and D.R. Tolan, *The temperature dependence of activity and structure for the most prevalent mutant aldolase B associated with hereditary fructose intolerance.* Archives of Biochemistry and Biophysics, 2002. **408**(2): p. 295-304. DOI: 10.1016/S0003-9861(02)00546-5.

5. Schuster, R. and H.G. Holzhutter, *Use of mathematical models for predicting the metabolic effect of large-scale enzyme activity alterations. Application to enzyme deficiencies of red blood cells.* Eur J Biochem, 1995. **229**(2): p. 403-18.
6. Rider, C.C. and C.B. Taylor, *Enolase isoenzymes in rat tissues. Electrophoretic, chromatographic, immunological and kinetic properties.* Biochim Biophys Acta, 1974. **365**(1): p. 285-300.
7. Meek, D.W. and H.G. Nimmo, *Effects of phosphorylation on the kinetic properties of rat liver fructose-1,6-bisphosphatase.* Biochem J, 1984. **222**(1): p. 125-30.
8. Cori, C.F., S.F. Velick, and G.T. Cori, *The combination of diphosphopyridine nucleotide with glyceraldehyde phosphate dehydrogenase.* Biochim Biophys Acta, 1950. **4**(1-3): p. 160-9.
9. Ryzlak, M.T. and R. Pietruszko, *Heterogeneity of Glyceraldehyde-3-Phosphate Dehydrogenase from Human-Brain.* Biochimica Et Biophysica Acta, 1988. **954**(3): p. 309-324. DOI: Doi 10.1016/0167-4838(88)90086-6.
10. Smith, C.M. and S.F. Velick, *The glyceraldehyde 3-phosphate dehydrogenases of liver and muscle. Cooperative interactions and conditions for functional reversibility.* J Biol Chem, 1972. **247**(1): p. 273-84.
11. Ponce, J., S. Roth, and D.R. Harkness, *Kinetic studies on the inhibition of glycolytic kinases of human erythrocytes by 2,3-diphosphoglyceric acid.* Biochim Biophys Acta, 1971. **250**(1): p. 63-74. DOI: 10.1016/0005-2744(71)90120-3.
12. Grossbard, L. and R.T. Schimke, *Multiple hexokinases of rat tissues. Purification and comparison of soluble forms.* J Biol Chem, 1966. **241**(15): p. 3546-60.
13. Bontemps, F., L. Hue, and H.G. Hers, *Phosphorylation of glucose in isolated rat hepatocytes. Sigmoidal kinetics explained by the activity of glucokinase alone.* Biochem J, 1978. **174**(2): p. 603-11.
14. Storer, A.C. and A. Cornishbowden, *Kinetics of Rat-Liver Glucokinase - Cooperative Interactions with Glucose at Physiologically Significant Concentrations.* Biochemical Journal, 1976. **159**(1): p. 7-14.
15. Van Schaftingen, E., *A protein from rat liver confers to glucokinase the property of being antagonistically regulated by fructose 6-phosphate and fructose 1-phosphate.* Eur J Biochem, 1989. **179**(1): p. 179-84.
16. Agius, L. and M. Peak, *Intracellular binding of glucokinase in hepatocytes and translocation by glucose, fructose and insulin.* Biochem J, 1993. **296 (Pt 3)**: p. 785-96.
17. Igarashi, Y., S. Kato, and K. Tada, *Kinetic properties of the glucose-6-phosphate transport system in rat hepatic microsomal membranes.* J Inherit Metab Dis, 1985. **8**(3): p. 153-4.
18. St-Denis, J.F., et al., *Glucose transport and glucose 6-phosphate hydrolysis in intact rat liver microsomes.* J Biol Chem, 1995. **270**(36): p. 21092-7.
19. Ciaraldi, T.P., R. Horuk, and S. Matthaei, *Biochemical and Functional-Characterization of the Rat-Liver Glucose-Transport System - Comparisons with the Adipocyte Glucose-Transport System.* Biochemical Journal, 1986. **240**(1): p. 115-123.
20. Arion, W.J. and R.C. Nordlie, *Liver Microsomal Glucose 6-Phosphatase, Inorganic Pyrophosphatase, and Pyrophosphate-Glucose Phosphotransferase. Ii. Kinetic Studies.* J Biol Chem, 1964. **239**: p. 2752-7.
21. Kelmer-Bracht, A.M., et al., *Kinetic properties of the glucose 6-phosphatase of the liver from arthritic rats.* Biochim Biophys Acta, 2003. **1638**(1): p. 50-6. DOI: S0925443903000413 [pii].
22. Tewari, Y.B., D.K. Steckler, and R.N. Goldberg, *Thermodynamics of isomerization reactions involving sugar phosphates.* J Biol Chem, 1988. **263**(8): p. 3664-9.
23. Zalitis, J. and I.T. Oliver, *Inhibition of Glucose Phosphate Isomerase by Metabolic Intermediates of Fructose.* Biochemical Journal, 1967. **102**(3): p. 753-&.
24. Edlund, G.L. and A.P. Halestrap, *The kinetics of transport of lactate and pyruvate into rat hepatocytes. Evidence for the presence of a specific carrier similar to that in erythrocytes.* Biochem J, 1988. **249**(1): p. 117-26.
25. Anderson, S.R., J.R. Florini, and C.S. Vestling, *Rat Liver Lactate Dehydrogenase. 3. Kinetics and Specificity.* J Biol Chem, 1964. **239**: p. 2991-7.
26. Prabhakaram, M. and S.N. Singh, *Effect of age on the crystalline rat liver lactate dehydrogenase.* Arch Gerontol Geriatr, 1986. **5**(1): p. 57-64. DOI: 0167-4943(86)90008-7 [pii].
27. Williamson, D.H., P. Lund, and H.A. Krebs, *The redox state of free nicotinamide-adenine dinucleotide in the cytoplasm and mitochondria of rat liver.* Biochem J, 1967. **103**(2): p. 514-27.
28. Indiveri, C., et al., *Kinetics of the Reconstituted Dicarboxylate Carrier from Rat-Liver Mitochondria.* Biochimica Et Biophysica Acta, 1989. **977**(2): p. 187-193. DOI: Doi 10.1016/S0005-2728(89)80070-2.
29. Raval, D.N. and R.G. Wolfe, *Malic Dehydrogenase .4. Ph Dependence of Kinetic Parameters.* Biochemistry, 1962. **1**(6): p. 1118-&. DOI: Doi 10.1021/Bi00912a024.

30. Crow, K.E., et al., *Rat liver cytosolic malate dehydrogenase: purification, kinetic properties, role in control of free cytosolic NADH concentration. Analysis of control of ethanol metabolism using computer simulation.* J Biol Chem, 1982. **257**(23): p. 14217-25.
31. Thorne, C.J.R., *Properties of Mitochondrial Malate Dehydrogenases.* Biochimica Et Biophysica Acta, 1962. **59**(3): p. 624-&.
32. Gelpi, J.L., et al., *Kinetic-Studies of the Regulation of Mitochondrial Malate-Dehydrogenase by Citrate.* Biochemical Journal, 1992. **283**: p. 289-297.
33. Wimhurst, J.M. and K.L. Manchester, *Some aspects of the kinetics of rat liver pyruvate carboxylase.* Biochem J, 1970. **120**(1): p. 79-93.
34. Wood, H.G., J.J. Davis, and H. Lochmuller, *The equilibria of reactions catalyzed by carboxytransphosphorylase, carboxykinase, and pyruvate carboxylase and the synthesis of phosphoendolpyruvate.* J Biol Chem, 1966. **241**(23): p. 5692-704.
35. Wilson, D.F., M. Erecinska, and V.L. Schramm, *Evaluation of the relationship between the intra- and extramitochondrial [ATP]/[ADP] ratios using phosphoenolpyruvate carboxykinase.* J Biol Chem, 1983. **258**(17): p. 10464-73.
36. Titheradge, M.A., R.A. Picking, and R.C. Haynes, *Physiological Concentrations of 2-Oxoglutarate Regulate the Activity of Phosphoenolpyruvate Carboxykinase in Liver.* Biochemical Journal, 1992. **285**: p. 767-771.
37. Colombo, G., G.M. Carlson, and H.A. Lardy, *Phosphoenolpyruvate Carboxykinase (Guanosine Triphosphate) from Rat-Liver Cytosol - Separation of Homogeneous Forms of Enzyme with High and Low Activity by Chromatography on Agarose-Hexane-Guanosine Triphosphate.* Biochemistry, 1978. **17**(25): p. 5321-5329. DOI: Doi 10.1021/Bi00618a001.
38. Adelman, R.C., F.J. Ballard, and Weinhous.S, *Purification and Properties of Rat Liver Fructokinase.* Journal of Biological Chemistry, 1967. **242**(14): p. 3360-&.
39. Jo, J.S., N. Ishihara, and G. Kikuchi, *Occurrence and Properties of 4 Forms of Phosphoenolpyruvate Carboxykinase in Chicken Liver.* Archives of Biochemistry and Biophysics, 1974. **160**(1): p. 246-254. DOI: Doi 10.1016/S0003-9861(74)80031-7.
40. Johnson, T.A. and T. Holyoak, *Increasing the Conformational Entropy of the Omega-Loop Lid Domain in Phosphoenolpyruvate Carboxykinase Impairs Catalysis and Decreases Catalytic Fidelity.* Biochemistry, 2010. **49**(25): p. 5176-5187. DOI: Doi 10.1021/Bi100399e.
41. Ballard, F.J., *Kinetic studies with cytosol and mitochondrial phosphoenolpyruvate carboxykinases.* Biochem J, 1970. **120**(4): p. 809-14.
42. Holyoak, T. and T. Nowak, *pH dependence of the reaction catalyzed by avian mitochondrial phosphoenolpyruvate carboxykinase.* Biochemistry, 2004. **43**(22): p. 7054-7065. DOI: Doi 10.1021/Bi049707e.
43. Palmieri, F., et al., *Kinetic Study of Tricarboxylate Carrier in Rat-Liver Mitochondria.* European Journal of Biochemistry, 1972. **26**(4): p. 587-&.
44. Uyeda, K., E. Furuya, and L.J. Luby, *The Effect of Natural and Synthetic D-Fructose 2,6-Bisphosphate on the Regulatory Kinetic-Properties of Liver and Muscle Phosphofructokinases.* Journal of Biological Chemistry, 1981. **256**(16): p. 8394-8399.
45. Van Schaftingen, E., et al., *Control of liver 6-phosphofructokinase by fructose 2,6-bisphosphate and other effectors.* Proc Natl Acad Sci U S A, 1981. **78**(6): p. 3483-6.
46. Reinhart, G.D. and H.A. Lardy, *Rat liver phosphofructokinase: kinetic activity under near-physiological conditions.* Biochemistry, 1980. **19**(7): p. 1477-84.
47. Sakakibara, R., S. Kitajima, and K. Uyeda, *Differences in kinetic properties of phospho and dephospho forms of fructose-6-phosphate, 2-kinase and fructose 2,6-bisphosphatase.* J Biol Chem, 1984. **259**(1): p. 41-6.
48. Vanschaftingen, E., D.R. Davies, and H.G. Hers, *Fructose-2,6-Bisphosphatase from Rat-Liver.* European Journal of Biochemistry, 1982. **124**(1): p. 143-149.
49. Cornell, N.W., M. Leadbetter, and R.L. Veech, *Effects of free magnesium concentration and ionic strength on equilibrium constants for the glyceraldehyde phosphate dehydrogenase and phosphoglycerate kinase reactions.* J Biol Chem, 1979. **254**(14): p. 6522-7.
50. Krietsch, W.K. and T. Bucher, *3-phosphoglycerate kinase from rabbit sceletal muscle and yeast.* Eur J Biochem, 1970. **17**(3): p. 568-80.
51. Fritz, P.J. and E.L. White, *3-Phosphoglycerate kinase from rat tissues. Further characterization and developmental studies.* Biochemistry, 1974. **13**(3): p. 444-9.
52. Mulquiney, P.J., W.A. Bubb, and P.W. Kuchel, *Model of 2,3-bisphosphoglycerate metabolism in the human erythrocyte based on detailed enzyme kinetic equations: in vivo kinetic characterization of 2,3-*

bisphosphoglycerate synthase/phosphatase using 13C and 31P NMR. Biochem J, 1999. **342 Pt 3**: p. 567-80.
53. Calvin, M.C., et al., *Human bisphosphoglycerate mutase expressed in E coli: purification, characterization and structure studies.* Biochimie, 1990. **72**(5): p. 337-43. DOI: 10.1016/0300-9084(90)90029-g.
54. Clarke, J.B., M. Birch, and H.G. Britton, *The equilibrium constant of the phosphoglyceromutase reaction.* Biochem J, 1974. **139**(3): p. 491-7.
55. Fundele, R. and W.K. Krietsch, *Purification and properties of the phosphoglycerate mutase isozymes from the mouse.* Comp Biochem Physiol B, 1985. **81**(4): p. 965-8.
56. Middleton, M.C. and D.G. Walker, *Comparison of the properties of two forms of pyruvate kinase in rat liver and determination of their separate activities during development.* Biochem J, 1972. **127**(4): p. 721-31.
57. van Berkel, T.J., J.K. Kruijt, and J.F. Koster, *Hormone-induced changes in pyruvate kinase. Effects of glucagon and starvation.* Eur J Biochem, 1977. **81**(3): p. 423-32.
58. Walker, P.R., et al., *Pyruvate-Kinase, Hexokinase, and Aldolase Isoenzymes in Rat-Liver Cells in Culture.* In Vitro-Journal of the Tissue Culture Association, 1972. **8**(2): p. 107-&.
59. Feliu, J.E., L. Hue, and H.G. Hers, *Hormonal control of pyruvate kinase activity and of gluconeogenesis in isolated hepatocytes.* Proc Natl Acad Sci U S A, 1976. **73**(8): p. 2762-6.
60. Titheradge, M.A. and H.G. Coore, *Mitochondrial Pyruvate Carrier, Its Exchange Properties and Its Regulation by Glucagon.* Febs Letters, 1976. **63**(1): p. 45-50. DOI: Doi 10.1016/0014-5793(76)80191-3.
61. Halestrap, A.P., *The mitochondrial pyruvate carrier. Kinetics and specificity for substrates and inhibitors.* Biochem J, 1975. **148**(1): p. 85-96.
62. Lee, E.W., et al., *Purification and Properties of Liver Triose Phosphate Isomerase.* Biochimica Et Biophysica Acta, 1971. **242**(1): p. 261-&.
63. Das, B. and S.K. Srivastava, *Purification and properties of aldose reductase and aldehyde reductase II from human erythrocyte.* Arch Biochem Biophys, 1985. **238**(2): p. 670-9. DOI: 10.1016/0003-9861(85)90213-9.
64. Lindstad, R.I., L.F. Hermansen, and J.S. McKinley-McKee, *The kinetic mechanism of sheep liver sorbitol dehydrogenase.* Eur J Biochem, 1992. **210**(2): p. 641-7. DOI: 10.1111/j.1432-1033.1992.tb17465.x.
65. Parks, R.E., E. Bengershom, and H.A. Lardy, *Liver Fructokinase.* Journal of Biological Chemistry, 1957. **227**(1): p. 231-242.
66. Lehninger, A.L., J. Sice, and E.V. Jensen, *Effect of Substrate Structure on the Aldolase Equilibrium.* Biochimica Et Biophysica Acta, 1955. **17**(2): p. 285-287. DOI: Doi 10.1016/0006-3002(55)90368-1.
67. Cox, T.M., M.W. Odonnell, and M. Camilleri, *Isolation and Characterization of a Mutant Liver Aldolase in Adult Hereditary Fructose Intolerance - Identification of the Enzyme Variant by Radioassay in Tissue Biopsy Specimens.* Journal of Clinical Investigation, 1983. **72**(1): p. 201-213. DOI: Doi 10.1172/Jci110958.
68. Gefflaut, T., et al., *Class I aldolases: Substrate specificity, mechanism, inhibitors and structural aspects.* Progress in Biophysics & Molecular Biology, 1995. **63**(3): p. 301-340. DOI: Doi 10.1016/0079-6107(95)00008-9.
69. Frandsen, E.K. and N. Grunnet, *Kinetic Properties of Triokinase from Rat Liver.* European Journal of Biochemistry, 1971. **23**(3): p. 588-&.
